# Barriers and enablers to routine register data collection for newborns and mothers: EN-BIRTH multi-country validation study

**DOI:** 10.1186/s12884-020-03517-3

**Published:** 2021-03-26

**Authors:** Donat Shamba, Louise T. Day, Sojib Bin Zaman, Avinash K. Sunny, Menna Narcis Tarimo, Kimberly Peven, Jasmin Khan, Nishant Thakur, Md. Taqbir Us Samad Talha, Ashish K.C., Rajib Haider, Harriet Ruysen, Tapas Mazumder, Md. Hafizur Rahman, Md. Ziaul Haque Shaikh, Johan Ivar Sæbø, Claudia Hanson, Neha S. Singh, Joanna Schellenberg, Lara M. E. Vaz, Jennifer Requejo, Joy E. Lawn, EN-BIRTH Study Group, Qazi Sadeq-ur Rahman, Ahmed Ehsanur Rahman, Tazeen Tahsina, Sojib Bin Zaman, Shafiqul Ameen, Tanvir Hossain, Abu Bakkar Siddique, Aniqa Tasnim Hossain, Tapas Mazumder, Jasmin Khan, Taqbir Us Samad Talha, Rajib Haider, Md. Hafizur Rahman, Anisuddin Ahmed, Shams El Arifeen, Omkar Basnet, Avinash K. Sunny, Nishant Thakur, Rejina Gurung, Anjani Kumar Jha, Bijay Jha, Ram Chandra Bastola, Rajendra Paudel, Asmita Paudel, K. C. Ashish, Nahya Salim, Donat Shamba, Josephine Shabani, Kizito Shirima, Menna Narcis Tarimo, Godfrey Mbaruku, Honorati Masanja, Louise T. Day, Harriet Ruysen, Kimberly Peven, Vladimir Sergeevich Gordeev, Georgia R. Gore-Langton, Dorothy Boggs, Stefanie Kong, Angela Baschieri, Simon Cousens, Joy E. Lawn

**Affiliations:** 1 Department of Health Systems, Impact Evaluation and Policy, Ifakara Health Institute, Dar es Salaam, Tanzania; 2 Centre for Maternal, Adolescent, Reproductive & Child Health (MARCH), London School of Hygiene & Tropical Medicine, Keppel St, London, UK; 3 Maternal and Child Health Division, International Centre for Diarrhoeal Disease Research, Bangladesh (icddr,b), Dhaka, Bangladesh; 4 Golden Community, Kathmandu, Nepal; 5 Florence Nightingale Faculty of Nursing, Midwifery & Palliative Care, King’s College London, London, UK; 6 International Maternal and Child Health, Department of Women’s and Children’s Health, Uppsala University, Uppsala, Sweden; 7 Department of Informatics, University of Oslo, Oslo, Norway; 8 Global Public Health Karolinska Institutet, Stockholm, Sweden; 9 International Programs, Population Reference Bureau, Washington DC, USA; 10 UNICEF Headquarters, New York, USA

**Keywords:** Birth, Maternal, Newborn, Coverage, Facility registers, Indicators, Data quality, Health management information systems

## Abstract

**Background:**

Policymakers need regular high-quality coverage data on care around the time of birth to accelerate progress for ending preventable maternal and newborn deaths and stillbirths. With increasing facility births, routine Health Management Information System (HMIS) data have potential to track coverage. Identifying barriers and enablers faced by frontline health workers recording HMIS source data in registers is important to improve data for use.

**Methods:**

The EN-BIRTH study was a mixed-methods observational study in five hospitals in Bangladesh, Nepal and Tanzania to assess measurement validity for selected *Every Newborn* coverage indicators. We described data elements required in labour ward registers to track these indicators. To evaluate barriers and enablers for correct recording of data in registers, we designed three interview tools: a) semi-structured in-depth interview (IDI) guide b) semi-structured focus group discussion (FGD) guide, and c) checklist assessing care-to-documentation. We interviewed two groups of respondents (January 2018–March 2019): hospital nurse-midwives and doctors who fill ward registers after birth (*n* = 40 IDI and *n* = 5 FGD); and data collectors (*n* = 65). Qualitative data were analysed thematically by categorising pre-identified codes. Common emerging themes of barriers or enablers across all five hospitals were identified relating to three conceptual framework categories.

**Results:**

Similar themes emerged as both barriers and enablers. First, register design was recognised as crucial, yet perceived as complex, and not always standardised for necessary data elements. Second, register filling was performed by over-stretched nurse-midwives with variable training, limited supervision, and availability of logistical resources. Documentation complexity across parallel documents was time-consuming and delayed because of low staff numbers. Complete data were valued more than correct data. Third, use of register data included clinical handover and monthly reporting, but little feedback was given from data users.

**Conclusion:**

Health workers invest major time recording register data for maternal and newborn core health indicators. Improving data quality requires standardised register designs streamlined to capture only necessary data elements. Consistent implementation processes are also needed. Two-way feedback between HMIS levels is critical to improve performance and accurately track progress towards agreed health goals.

**Supplementary Information:**

The online version contains supplementary material available at 10.1186/s12884-020-03517-3.

## Key findings

**Table:** 

**What is known and what is new about this study?** • Routine facility register data recorded in Health Management Information Systems (HMIS) in low- and middle-income countries (LMICs) provide an opportunity to close data gaps for tracking coverage of care at birth. Although around four-fifths of the world’s births are now in facilities, labour ward register data are currently under-used and under-studied. Specifically, few studies have examined barriers and enablers for recording high quality routine maternal and newborn data, or on the use of labour and delivery ward registers. • EN-BIRTH was the first multi-country, mixed-methods study to assess validity of register-recorded maternal and newborn coverage indicators. In the three study countries, we found register coverage measurement accuracy varied, even between hospitals in the same country using the same registers. • Hence to assess barriers and enablers for health workers to record data in labour ward registers, we interviewed health workers (*n* = 72) and EN-BIRTH research data collectors (*n* = 65) across the five hospitals. **What did we find and what does it mean?** • **DESIGN** of national labour ward registers varied between the study countries, capturing between 35 and 58 data elements, duplicative with other recoding in other documents. Coverage indicators of interest (uterotonics, early initiation of breastfeeding and neonatal bag-mask-ventilation) are recorded in registers in Bangladesh and Tanzania but not in Nepal. Standardisation of registers and linkage of these registers to digital HMIS is urgently needed for global tracking. Registers also need local ownership to streamline with local facility documentation requirements, this is critical to reduce burden on frontline health workers. • **FILLING** processes of routine registers are not systematically implemented within or between countries. Completeness was more highly valued than accuracy. Consistency and accuracy could be promoted by training and supportive supervision to realize the potential of this data source. • **USE** of register data are impeded by lack of trust in its quality. Promotion of the importance of health facility data for clinical quality improvement, and monitoring is needed to improve data quality and use. Feedback from data users at supervisor/manager and district levels could increase the value frontline health workers attribute to these data and promote their use at the place of care. **What next and research gaps?** • Routine labour ward register data can be used now to contribute vital data around the time of birth. Implementation research is required on interventions to standardise labour ward register designs, and the processes for filling them with regular data quality review. Such research could test an improvement package to include a two-way data flow system up from labour ward registers into HMIS, and feedback returning to the facility.	

## Background

Data gaps to track care around the time of birth in low- and middle-income country (LMIC) settings impede action towards goals to end more than 5 million deaths annually of newborns, stillbirths and women [1–4]. Although > 80% of the world’s births occur in facilities [5], routine records are under-utilised as a data source for maternal and newborn care. The *Every Newborn* Action Plan (ENAP), agreed by all United Nations member states and > 80 development partners, includes an ambitious measurement improvement roadmap with an urgent focus to improve measurement around the time of birth, especially routine Health Management Information System (HMIS) data [6]. Sustainable Development Goal 17 “Revitalise the global partnership for sustainable development” includes a specific target to increase the availability of high-quality, timely and reliable data [7]. Population-based surveys remain a major source of maternal and child health data in LMIC [8–10]. Such household surveys— e.g. the Demographic and Health Surveys (DHS) Program [11] and Multiple Indicator Cluster Surveys (MICS) [12, 13]— collect information regarding births over the preceding 2 to 5 years, thus are not designed to tracking progress on a month-to-month, or year-to-year basis [1, 14–16].

Routine HMIS data, in contrast, have potential to be available more regularly and used for more timely action by health workers, facility/district managers and policy makers [17]. The expansion of digital platforms e.g. District Health Information Software 2 (DHIS-2) in LMICs in recent years has increased awareness of the potential of HMIS to improve data availability at the subnational level and above [18]. Whilst household surveys are designed to be representative of populations, as institutional births rise, facility HMIS data is becoming increasingly useful. However, HMIS data quality has historically been considered poor, so increasing data quality and trust are essential [19, 20]. Studies in LMICs have shown how data use positively impacts quality of care and helps strengthen health systems [21, 22]. The performance of routine information system management (PRISM) framework illustrates the multiple factors (organisational, technical and behavioural) that influence data quality and information use (Table 1) [23, 24]. Routine register data are usually the source for HMIS facility data. Paper registers are books, typically located on a hospital ward; they contain a limited number of data elements as a parallel and usually duplicate system to individual patient case notes. Health workers record each admitted individual women/newborn on one row in the register with data regarding care practices and interventions in columns allotted either for “specific” data elements (e.g. bag-mask-ventilation) or “non-specific” data elements (e.g. other details). Previous studies have assessed availability and completeness of data elements for maternal and newborn coverage indicators in routine registers [25, 26]. Data for local and higher health system use in HMIS are typically aggregated from registers monthly, using paper tally sheets and/or summary forms. Data culture within the health facility influences register data collection, analysis and use [27].

**Table 1:** Performance of routine information system management (PRISM) conceptual framework components

Type	Category	Content
**INPUTS** RHIS Determinants	**Technical Factors**	Complexity of reporting forms, procedures
		HIS design
		Computer Software
		Information technology complexity
	**Organisational Factors,**	Governance
		Planning
		Training
		Supervision
		Quality
		Finance
		Promotion of culture of information
		Availability of resources
	**Behavioural factors**	Level of knowledge of content of HIS forms
		Data quality checking skills
		Problem solving for HIS tasks
		Competence in HIS tasks
		Confidence levels for HIS tasks
		Motivation
		Demand
**PROCESS** steps	**RHIS processes**	Data collection
		Data transmission
		Data processing
		Data analysis
		Data quality check
		Feedback
**OUTPUT** desired	**Improved RHIS performance**	Data quality/information use
**OUTCOME** desired	**Improved Health System performance**	
**IMPACT** desired	**Improved health status**	Improved health status

References: PRISM framework and monitoring framework for ending preventable maternal mortality [23, 24]

The *Every Newborn* – Birth Indicators Research Tracking in Hospitals (EN-BIRTH) study was a mixed methods observational study in three countries (Tanzania, Bangladesh and Nepal). EN-BIRTH aimed to assess measurement validity of newborn and maternal indicators for routine facility-based tracking of coverage, quality of care, and outcomes (21). Indicators were selected based on criteria outlined in global frameworks [6, 28, 29]. The EN-BIRTH validation assessment reported finding register-recorded coverage accuracy varied by indicator and by hospital [30].

## Objectives

This paper is part of a supplement based on the EN-BIRTH multi-country validation study, *‘Informing measurement of coverage and quality of maternal and newborn care’*. The purpose of this paper is to explore general barriers and enablers for health workers to record high-quality (complete and accurate) data in labour ward registers only. Data recorded in registers in neonatal and kangaroo mother care wards are explored in other papers in the supplement [31, 32]. This paper has three objectives:

1. **Describe the STRUCTURE OF ROUTINE LABOUR WARD REGISTERS** for measurement of coverage of key maternal and newborn health intervention indicators.
2. **Identify BARRIERS AND ENABLERS** for health workers to record and use labour ward register data for measurement of coverage of key maternal and newborn interventions.
3. **Explore the PROCESSES** of labour ward health care provision and register documentation including flow and sequence, by health workers for key maternal and newborn interventions at birth.

## Methods

### Study sites and overview

EN-BIRTH study was conducted in five public hospitals in three high-burden mortality countries: Maternal and Child Health Training Institute, Azimpur and Kushtia District Hospital in Bangladesh (BD), Pokhara Academy of Health Sciences in Nepal (NP), and Temeke Regional Hospital and Muhimbili National Referral Hospital in Tanzania (TZ) (Additional file 1). These comprehensive emergency obstetric and newborn care (CEmONC) hospitals were selected since they provided the interventions of interest across several different wards. Labour ward register findings for three indicators (uterotonics to prevent post-partum haemorrhage, early initiation of breastfeeding and neonatal bag-mask-ventilation) will be reported in this manuscript; other ward findings are reported in separate manuscripts [31–33]. The multi-partner research team co-designed the tools and collected data from January 2018 to March 2019.

### Objective 1: Structure of routine labour ward registers

We reviewed the design structure for labour ward registers to summarise: total number of data elements captured; selected indicator data elements column name, column type (specific or non-specific) and how the column was completed if the intervention was either given or not given.

### Objective 2: Barriers and enablers to record and use register data

The research team, using a literature review, identified the PRISM conceptual framework (Table 1) and used these constructs to design guides for semi-structured in-depth interviews (IDI) and for focus group discussions (FGD) (Additional file 2). The guides explore routine labour ward register documentation in general, with specific open-ended questions about selected indicators (Additional files 3, 4) [33]. Tools were developed in English, translated to local languages (Bengali, Nepali and Swahili), then piloted, revised and back-translated into English.

#### Respondents and data collection

We purposively selected two groups of respondents: (i) Health workers (nurses/midwives/doctors) from the study hospitals routinely caring for women/newborns and are responsible for recording in ward registers; and (ii) EN-BIRTH study researchers (clinical observers, data extractors and supervisors) who were present for more than 9 months on the study site wards, for an external perspective on the register documentation process [33].

At least two IDIs were conducted in each site for each category of respondent. The sample size for the interviews was determined using saturation sampling: additional respondents were interviewed until no new information was learnt by the investigators in each site. One FGD including at least one health worker from each ward was added for triangulation. Data were collected by experienced qualitative researcher co-authors in two phases: January–June 2018 for EN-BIRTH study data collectors and January–March 2019 for EN-BIRTH study hospital health workers. Interviews were conducted in local languages in a private room and audio recorded after obtaining informed participant consent.

#### Data management and analysis

Data transcription, translation into English, codebook design and analysis were carried out by the same co-authors involved with tool design and data collection after all data had been collected. All transcripts were read multiple times by team members prior to developing the codebook for familiarization. A coding template in NVivo software version 12 [34] was jointly developed based on the PRISM framework (Additional files 5, 6) and the codebook. Framework analysis was used to support comparing, and to differentiate between IDI and FGD findings [35]. Two coders from each country team coded the same 2–4 interviews and compared results. Any discrepancies were discussed, which increased inter-coder reliability [36]. Differences were reconciled through discussion or involvement of another team member, and single individuals coded remaining transcripts. The multi-country team reconciled coding issues on weekly calls and the codebook was modified where necessary.

For the health worker-register interface, the EN-BIRTH team created a framework based around three categories: register design, register filling and register use. We applied this conceptual framework to identify emerging themes across all sites. Two analysis workshops and multiple multi-country calls were held to agree upon the main themes emerging from the IDIs and FGDs, and to synthesise the findings. The consolidated criteria for reporting qualitative research (COREQ) checklist guidelines were followed throughout (Additional file 7) [37].

### Objective 3: Processes of care and documentation including flow and sequence

To identify how health care provision and labour ward register documentation relate to one another on labour ward, we designed a third tool called the “care-to-documentation checklist” (Additional file 8). This tool captured the process, flow and sequence of recording data in registers by selected indicators: which health worker cadre usually/sometimes provides the care; which cadre records the care; what is the order of documentation in labour ward documents (among register, patient notes, drug charts, partograph); what is the estimated time in minutes between intervention given and documentation. These close-ended questions were asked by the researcher to respondents, immediately after their IDI (but not to FGD respondents). The checklist data were entered on Excel and proportions and sequence were analysed in R version 3.6.1 [38].

## Results

### Objective 1: Structure of routine labour ward registers

We identified two types of registers on the labour wards: formal pre-printed and informal hand-written registers, which are typically facility-specific for programme or quality improvement purposes (Additional file 9). All study hospitals used nationally developed, formal paper-based registers; in Bangladesh, a national register was introduced during the early phase of the study, replacing previously existing, hospital-specific ones. In Muhimbili TZ, the informal “Perinatal Research Register” has been in continuous use for more than 20 years [39]. In Temeke TZ, one nurse-midwife was assigned to send summary data every day from the register to HMIS and had no other clinical responsibilities. The total number of data elements captured in formal register columns was: 58 in Bangladesh, 35 in Nepal and 48 in Tanzania (Table 2). One data element was captured per column in the register in Tanzania, but more than one in some register columns in Bangladesh and Nepal. Data elements needed as numerators for the three selected coverage indicators were captured in the Bangladesh and Tanzania registers but not in the Nepal register. In Bangladesh register columns were ticked when the intervention/practice was done and left blank when not done; in Tanzania, register columns were filled with yes/no in Swahili, except for bag-mask-ventilation, which was completed with a numerical code (Additional file 10).

**Table 2:** Ward routine register designs capturing selected newborn and maternal indicators, EN-BIRTH study

Register design	BD - Azimpur		BD - Kushtia		NP - Pokhara	TZ - Temeke	TZ - Muhimbili	
	Tertiary		District		Regional	Regional	National	
**Labour and Delivery Ward**								
**Register name**	Delivery register	EmONC register	Delivery register	EmONC register	Maternity Register	Delivery book	Delivery book	Perinatal research register
**Register format**	Original hospital	Revised national	Original hospital	Revised national	National	National	National	Additional research
**Number of data elements**	25	58	24	58	35	48	48	47
**Number of columns:**								
total	20	45	18	45	32	48	48	39
for uterotonics	1	1	1	1	0	2	2	2
for early breastfeeding	0	1	0	1	0	2	2	2
for neonatal resuscitation	0	1	0	1	0	1	1	1

Details regarding selected indicators in Additional file 10

Note: register designs may record more than 1 data element per column. BD = Bangladesh, NP = Nepal, TZ = Tanzania

### Objective 2: Barriers and enablers to record and use register data

A total of 72 health workers (62 nurse-midwives and 10 medical doctors) and 65 data collectors were interviewed for this study (Table 3); background characteristics of participants are shown in Additional file 11.

**Table 3:** Summary of research methods assessing barriers and enablers to labour ward register documentation, EN-BIRTH study

Method	Description of the method	Duty ward	Responsibility	Selected indicator documented explored
**Heath workers:**				
**a) In-depth interviews and** **c) care-documentation checklist**	Nurses/midwives (*n* = 3 per hospital, total *n* = 15)	Labour and Delivery	Care for patient and document	• Uterotonics to prevent PPH • Early initiation of breastfeeding • Neonatal bag mask ventilation
	Doctors (*n* = 1 per hospital, total *n* = 5)	Labour and Delivery included	Care for patient and document	All selected indicators
**b) Focus Group Discussion**	Nurses/midwives from each ward (*n* = 1 FGD per hospital, total *n* = 5)	Labour and Delivery included	Care for patient and document	All selected indicators
**EN-BIRTH data collectors:**				
**a) In-depth interviews and** **c) care-documentation checklist**	Data Trackers (*n* = 3–4 per hospital, total *n* = 19)	Registered patient at start of study	Observed care process and some content of documentation	Not applicable
	Clinical observers (*n* = 4–8 per hospital, total *n* = 24)	All wards	Observed care process but not content of documentation	All selected indicators
	Data Verifier/Extractor (*n* = 1–4 per hospital, total *n* = 13)	All wards	Extracted data from registers and patient notes for EN-BIRTH study	All selected indicators
	Supervisors (*n* = 1–2 per hospital, total *n* = 9)	All wards	Observed process and extracted data from registers and patient notes	All selected indicators

Further details of respondents from all wards in Additional file 2

**Fig. 1:**
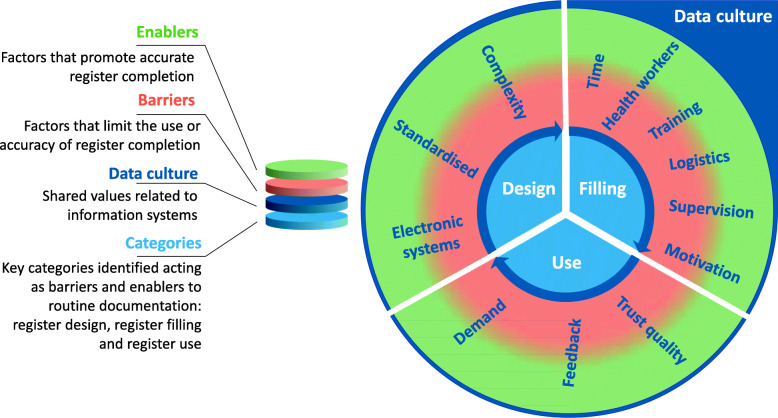
Barriers and enablers to routine recording of coverage indicators in labour ward registers, EN-BIRTH study. The transition from red (barrier) to green (enabler) serves as a reminder that most factors identified by participants could serve as either a barrier or enabling factor depending on the facility-level resources and management

As shown in Fig. 1, participants reported that these common themes could either serve as barriers or enablers to recording and using register data. The themes are shown radiating from the conceptual framework to illustrate how these themes were described as influencing one another and the hospital data culture. Each theme is summarised in turn below.

#### Register design

Three themes emerged:

##### Complexity

The labour ward registers were described as complex by many respondents in Tanzania and Bangladesh:

*“It is complicated somehow, first it is large and that book [register] contains a lot of details to be filled although all of them are important … .”*

-IDI, L&D Nurse-midwife, Muhimbili TZ

Additionally, the data elements recorded in the formal labour ward register need to be duplicated in multiple documents (e.g. informal registers, patient notes), as complex registers form part of a documentation system that is not streamlined and is burdensome:

*“We need to do the same documentation, again and again in three to four different places, which needs us to give a lot of time.”*

-IDI, L&D Nurse-midwife, Azimpur BD

##### Standardisation with necessary data elements

Health workers from the Nepal and Bangladesh sites acknowledged all the data elements they needed were captured. However, in Tanzania, not all data elements needed to complete monthly reporting forms for HMIS were in the labour ward registers:

*“I enter entire patient’s information … and I sometimes have to add some columns where I can include some data that I know is important and should be written to help me with my end of the month report. So, if I were to just follow the register it means some data could be missed and that’s the challenge that I encounter.”*

-FGD, Nurse-midwife, Muhimbili TZ

##### Paper or electronic

All five hospitals were using paper-based registers, but respondents mentioned forthcoming transition to electronic platforms, which were anticipated to be desirable, to save time, and to improve data completeness, availability, and storage:

*“Documentation till today is done in traditional way. However, writing that every day, is time loss. Further, if we had computerised system, it would have been very better, it could last for later.”*

-IDI, L&D Nurse-midwife, Pokhara NP

Yet many respondents expressed their need for computer training, some suggested extra staff would need to be recruited to manage digitised registers:

*“To operate the computer for documentation, we need both manpower and proper training. For example, if we had three more staffs in this ward, two staffs will work for caring the patient and the other one will engage with documentation and can handle the computer. It will allow us to perform other things more easily.”*

-IDI, L&D Nurse-midwife, Azimpur BD

#### Register filling

Six themes emerged:

##### Health worker responsibility

In all five hospitals nurse-midwives alone owned the task of labour ward register recording described as within their current nursing role. Data quality responsibility was perceived to be better when the same nurse-midwife providing the care documented in the register:

*“For effective recording and reporting, the one who provides the care should herself do the documentation and then only it is complete and proper. A third person asked to document is not proper – there will be missing in recording and reporting. Manpower should be sufficient so the one who does the care should only perform recording and reporting.”*

-FGD, Nurse-midwife, Pokhara NP

However, task shifting of documentation to other actors was highly valued by several respondents, although difficult to obtain, especially during night shifts:

*“It is super difficult to get support from students even the intern doctor and the trainee nurses don’t help us in documenting the information in register.”*

-FGD, Nurse-midwife, Kushtia BD

##### Training for competence

Respondents from Nepal described attending a short training on register filling as an enabling factor for register data recording. Tanzanian respondents stated they had been shown “on the job” how to fill the register and the lack of specific formal training or instructions for register filling was a barrier to documentation. In Bangladesh, only computer training had been received:

*“We have not got any training related to register fill up. We were given only an orientation on computer but couldn’t learn anything. It was too short i.e. 2 to 3 days.”*

-IDI, L&D Nurse-midwife, Azimpur BD

##### Time required to document

Respondents expressed the large amount of time spent on documentation in general, even in the Nepal site with the lighter register design:

*“If we work 7 to 8 hours duty, it usually takes around 3 hours to do documentation.”*

-IDI, Nurse-midwife, Pokhara NP

*“In a period of 8 hours of my shift, if I have a large number of patients, I may spend more time in documentation than the time I spend in attending the patients.”*

-IDI, L&D Nurse-midwife, Muhimbili TZ

In all three countries, respondents related the time challenge of completing registers to the availability of the health workforce:

*“Our main difficulty to fill up the register appropriately, is shortage of manpower. We have to suffer a lot to do quality documentation due to short of manpower.”*

-IDI, L&D Nurse-midwife, Azimpur BD

The tension between being too busy to always document immediately after care led to lower quality data:

*“You find you are having say three patients and they all need care, you will start with the first one, after that you can’t do the documentation, you will have to attend the second and the third, now as you go for documentation it will be difficult to remember exactly figures or details, for example it is difficult to remember exactly the time for each of them so, you will have to estimate, maybe if you have enough staff, one does the attending and another do the documentation.”*

-IDI, Nurse-midwife, Muhimbili TZ

##### Logistical resources needed

New registers were usually available but sometimes the stock were locked in stores. Pens were only available in some hospitals:

*“There is still a challenge of resources, for instance now we are asked to document but they don’t think if pens are provided, instead you have to buy yourself. You are supposed to write … .and there are some things which I would like to write them if they would provide me with tools. Honestly resource is very challenging”*

-FGD, Nurse-midwife, Muhimbili TZ

The organisation of the large registers laying on a table at the nursing station were described as a logistical barrier by some respondents:

*“When she is done she will go to the nursing station to do her documentation in register book, then fills the midwifery book, the books are in different places and are far from the patient or the delivery room.”*

-IDI, EN-BIRTH Data Collector, Muhimbili TZ

##### Supervision for data quality

Supervision of register filling processes was acknowledged to be an important enabler to register filling by most respondents, yet was not occurring regularly in every hospital:

*“We never had any sorts of supervision about the documentation.”*

-IDI, L&D Nurse-midwife, Azimpur BD

*“The only things that displays the work of health workers are the documentations … important for supervisors as well. If we show them the recorded data, they get to advise us about the errors and whether it [register] is complete or not. So it becomes important in supervision as well.”*

-IDI, L&D Nurse-midwife, Pokhara NP

Register documentation supervision was expressed as being linked to data quality:

*“They normally come to verify their data on register books and if there is any problem, they tell you that here you are supposed to do this and that. This is how is being done … It is educative system because if she criticise you she must explain to you.”*

-FGD, Nurse-midwife, Temeke TZ

Many respondents expressed that completeness was important and the need to “fill the gaps” in registers:

*“There is a big delivery book which has headings therefore, you can’t skip even a single box all of them must be filled.”*

-FGD Nurse-midwife, Muhimbili TZ

##### Motivation

Appreciation from supervisors was articulated by one respondent as an important motivator, and was also linked with higher quality documentation:

*“We receive praise, when everything (related to documentation) is good and it works as a motivation to continue documentation with care.”*

-IDI, L&D Nurse-midwife, Azimpur BD

By contrast, many health workers noted the lack of acknowledgement and/or recognition served as a motivational barrier for high quality register recording:

*“There is not any formal award or recognition like that. Instead we get scolded if it’s left. We are not appreciated for writing.”*

-FGD, Nurse-midwife, Pokhara NP

#### Register use

Three themes emerged regarding perceived register data utility:

##### Demand for data

Respondents expressed varied register data demands as enablers. Nurse-midwife respondents mainly described how they themselves used the data for patient handover:

*“We are documenting because even nursing itself is a continuous process … so if you did not document, the other nurse will not know where you ended, so documentation is still very important.”*

-FGD, Nurse-midwife, Temeke TZ

The same register data were used by supervisors for management decisions:

*“Even the hospital itself insists so much on documentation... if you don’t document, sometimes it becomes very difficult for the management to get revenue to know how many people should get what medicine, you have to document on health insurance and normal patients.”*

-FGD, Nurse-midwife, Temeke TZ

In Nepal, a doctor respondent expressed that data were used in research and for indicators:

*“We also have doctors and students utilising the data. It is used for the research and general information. We create health indicators and send to central level and they also create national health indicators. And the ultimate goal for all is to know how the health indicators are. It helps to do planning accordingly.”*

*-*IDI, L&D Doctor, Pokhara NP

##### Feedback to health workers

Provision of feedback from HMIS users of register data to those who had collected the data was perceived to be an enabler; however, respondents said feedback hardly ever happened:

*“I haven’t got any feedback from them (HMIS) about documentation. There sits monthly meeting in hospital with data people. We don’t usually participate in that meeting.”*

-IDI L&D, Nurse-midwife, Azimpur BD

*“It doesn’t come to us directly. We don’t have much information.”*

-FGD, Nurse-midwife, Pokhara NP

##### Trust in data quality

Some health worker respondents stated that lack of trust in register data quality was a barrier to the usefulness of register data:

*“Sometimes, variables are missing and when research needs to be done then it is not ineffective.”*

-IDI, L&D Doctor, Pokhara NP

*“There is hardly missing areas in the register- if we find some we try to collect the information either by asking the patient again or nurse who attended the delivery. Using good quality data are important to decision make.”*

-IDI, L&D Nurse, Kushtia BD

### Objective 3: Sequence of care and documentation

Analysis of the care-to-documentation checklist showed that the nurse-midwife who provided the intervention/practice usually also recorded in the labour ward register (Additional file 12). However, data collector respondents stated that health workers sometimes documented care provided by a colleague (Additional file 13). Among all documents to be filled, the labour ward register were described as the first to be completed in both Bangladeshi hospitals, but the order varied between first to third in the Tanzanian hospitals (Additional files 14, 15). The average estimated time between care provision and register documentation ranged from 10 to 28 min as reported by health workers and was 9 to 34 min based on data collectors’ report (Additional file 16).

## Discussion

EN-BIRTH study is the first LMIC multi-country assessment of barriers and enablers to labour ward register data recording. We add to previous research regarding barriers to routine facility data recording from antenatal clinics and HIV/AIDS programme data [1, 40, 41]. We found twelve consistent themes reported in all five hospitals across our conceptual framework of register design, filling and use. Figure 1 depicts the interconnected relationship between register data use, register design, and register filling. The twelve themes identified within these categories were described as either enablers or barriers by respondents in the five hospitals. We postulate that the varying interaction of these themes in each study hospital contributed to the variation in accuracy in measurement of labour ward indicators as identified in the EN-BIRTH validation study [30]. These data practice themes act within, and likely contribute to, a wider hospital data culture of accepted and normative practices, which permits health workers to collect high-quality register data that can be trusted for use.

Improved HMIS performance is increasingly recognized as a priority to improve coverage and quality of care as described in the comprehensive PRISM framework, which demonstrates the many interacting constructs needed for high-quality data for use [23, 24]. This EN-BIRTH study used the PRISM constructs to explore the barriers and enablers to recording at the service user-register interface and for health workers. We found register design complexity and the burden of data collection were common across the study sites. The sheer volume of data elements captured in these national register designs was striking. Nepal had the lightest register design, yet captured 35 data elements, compared to 48 in Tanzania and 58 in Bangladesh. Notably, data elements more than doubled when national registers were introduced in Bangladesh. Yet labour ward registers did not always match monthly reporting requirements, necessitating nurse-midwives to use their own initiative and add columns to registers, or start informal registers, to capture required data. Complexity of documentation was described as encroaching upon the time health workers can dedicate to midwifery care. Our findings align with a study describing the balance between service provision and documentation practices in Uganda [42]. Several causes contribute to this high burden of register data collection, including a lack of coordination regarding which indicators (and contributing data elements) are selected for tracking, multiple reporting flows and additional data element capture to signal rigor or research [43]. Frontline health workers have dual responsibilities of providing care and documentation of that care. With the typically high user-to-staff ratios of facilities in many LMIC settings, urgent attention to reducing any unnecessary documentation would support efforts to improve quality of care by health workers for women and babies.

Filling of registers was not systematised or consistently supported by effective logistics and supplies, even non-availability of pens and registers was cited by some respondents. Bedside care provided by the health worker was documented in one register located on a table in the labour ward. The documentation was described as done within 30 min of the care practice/intervention whilst the health worker was still responsible for the women and her baby during the critical first hour after birth. The cumulative effect of distance between point of care and point of register documentation, simultaneous responsibilities of care and documentation for a large number of data elements to be recalled could account for both under and over-reporting of interventions, as found in the EN-BIRTH observational validation study [30].

Perceived value of labour ward register data by data users in these large CEmONC hospitals was a further cross-cutting issue that likely affects data quality [30]. Data-specific training was perceived by health workers as enabling, yet few had received in-service training on how to complete registers. Supportive supervision for register recording was not a priority, as described by both health workers and research data collectors. Data completeness was expressed as more highly valued compared to data accuracy by health workers and data collectors alike. This may be driven by column filling (completeness) being feasible to visualise in registers by health workers and supervisors, and thus a signal and symbol of professionalism [44]. Although notably in Bangladesh completeness for coverage numerators cannot be calculated, as registers are designed for columns to be left blank (true zero) when interventions are not performed.

Use of register data was valued by health workers for clinical care handover or other hospital use, however none of the nurse-midwife respondents who actually fill registers mentioned use for tracking coverage or impact of services at higher levels of the health system. Increasing demand for labour ward register data use is needed. Using register data at facility level to improve quality of care or to supervise performance was mentioned could link to priority setting and health unit management also at sub-national level. National data demand includes for strategic planning and policy. Health workers around the world invest considerable time documenting large volumes of data. Nurse-midwives deserve to be informed about the value of the data they collect for wider decision making, and to be appreciated for their work in collecting it.

Enabling environments are needed for health workers to provide care and are often measured as “service readiness” [45]. Similarly, enabling “data readiness” is necessary to promote high-quality register data to flow into HMIS. An integrated approach is needed to transform routine data on labour wards, taking into account the midwife’s dual role in care provision and data recording [20]. The information culture at the facility level and throughout the system is important. Decentralised data use in facilities may incentivise improving data quality [46, 47]. By increasing data visibility through feedback to frontline health workers about data use, data quality has been shown to improve in registers [14, 19, 22–24, 48–50]. However, a notable finding from our labour ward register study was the low level of two-way feedback loops between different levels of the data pyramid: nurse-midwives collecting register data and other data users higher up in the pyramid [51, 52].

Paper-based systems remain the norm in most LMIC labour wards, yet these often feed into digital systems [53]. However, care should be taken to not just digitise poor information systems. There has been rapid expansion of digital HMIS in LMIC with increased IT capability to improve data quality (automated checks, validation rules, visualizations, etc.) [1, 46, 47, 54]. Poor quality of care has been described as “too much too soon, too little too late” [55]. Similarly, in response to “too little data too late”, care is needed to avoid digitisation of routine data creating “too much data too soon”. Unless we turn our attention to reduce unnecessary data and improve reliability and quality of the register data, the value of digital HMIS data for clinical and programmatic decision making will not be realised. The risk is that labour ward routine register data will remain in a “vicious cycle of data quality”, if data are not trusted, they are not used. If data are not used, investment in data quality suffers, and data quality deteriorates even further. Thus, simultaneous action on both data use and data quality is necessary to break this cycle. In practice, this means increasing use of current labour ward register data, whilst investing in improving data quality. Current data quality reviews typically compare HMIS monthly reports using register data as the standard [56]. Innovative ways to routinely include assessment of the quality of the source register data are important to consider. Register data assessment can be linked to routine quality improvement initiatives that use routine data, such as maternal and perinatal death surveillance and response. Checking accuracy of register data quality compared to patient case notes during such perinatal audit meetings and involving health workers could be one effective way for feedback and linking quality of data with quality of care. Without focused action to improve routine data quality, tracking progress using HMIS data towards agreed Sustainable Development Goals and ENAP targets by 2030 will be suboptimal [53].

### Strengths and limitations

A strength of our study is multi-sites public hospitals in three high mortality burden LMICs. We used common tools that were co-designed by our team including the PRISM framework determinants. We interviewed health workers involved in the process themselves and, for an external perspective, EN-BIRTH research data collectors who had worked day and night on the labour ward for > 9 months. The use of open-ended and close-ended questionnaires allowed us to generate a broad range of common findings issues across sites. Our predetermined codes were based on the PRISM framework and all sites used NVivo in a collaborative analysis process.

However, our study also has limitations. There was a possible desirability bias by health workers, which might have led to either under- or over-reporting of the challenges faced. The “care-to-documentation checklist” dataset analysis was stratified by type of respondent (health worker and data collector), by indicator and by site. The qualitative data analysis presented in this paper identified common barriers and enablers for labour ward register recording across all indicators, using health worker and data collector responses together. Indicator-specific mixed-methods linked analyses will be presented in other linked papers to further explore subthemes and differences between cadres [57–62]. It was beyond the scope of this study for the EN-BIRTH data collectors to directly observe or measure the detailed process of register filling (e.g. time, logistics availability, supervision, use for reports). All hospitals were peri-urban CEmONC hospitals, which may limit generalizability to facilities at lower levels of the health system.

### Research for improving measurement

Further research is needed to explore barriers and enablers in other settings and at different levels of the health system to understand the broader relevance of the themes we identified. Our exploratory research identified twelve themes that could be used to design shorter tools for routine register data capture and use, a component of HMIS that is relatively under-represented in existing tools [27, 56]. Implementation research is required for all three components we identified regarding registers in our conceptual framework (design, filling, use). To enable national or district tracking of core indicators in HMIS, the priority data elements that are being harmonized at higher levels of the data pyramid will need to be included in register design [63, 64]. Register data element availability is necessary but not sufficient; more research is required to explore whether register layout, column labelling and cell coding affect data quality. For example, facilities might consider excluding blank cells from their register design, as blank cells may indicate a health procedure either “not recorded” (incomplete) or “not done”. Standardised register designs will require local ownership for adaptation, and testing of process, with considerable streamlining with other documentation, to reduce burden on frontline health workers. Research regarding improved register filling may focus on capability (capacity to engage in the register documentation), opportunity (factors that make the behaviour possible) and motivation (to energies and direct behaviour). Exploring flow of aggregated data from labour ward registers into HMIS is another gap requiring research regarding steps of aggregation. Several manual operations (e.g. manual counting, filling paper summary/tally forms, digital data entry) may reduce data quality significantly [65]. Finally, perspectives of data users beyond the patient-health worker-register interface are critical. Yet, to date, there has been little investment in improving routine register data quality to maximize the potential of this underused and widely available data source around the time of birth.

## Conclusion

With more than 80% of the world’s births in facilities, labour ward register data have an unrealised potential to track core indicators in facilities and higher up the health system. Our multi-country study found multiple opportunities to improve the data and the use of data: standardised design, consistent filling processes and enabling two-way feedback between different levels of the health system data pyramid. Overcoming these barriers would enable frontline health workers, especially midwives, to be valued for the register data they collect, to improve data quality and importantly to use those data to improve quality of care for the women and babies they care for.

## Supplementary Information

**Additional file 1.** National context and number of births in EN-BIRTH study hospital.

**Additional file 2.** Summary of qualitative research methods to assess barriers and enablers to labour/newborn ward register documentation, EN-BIRTH study.

**Additional file 3.** Health Worker study guides in-depth interview (IDI) focus group discussion (FGD), EN-BIRTH study.

**Additional file 4.** Data Collector study guides in-depth interview (IDI), EN-BIRTH study.

**Additional file 5.** Codebook Health Workers, EN-BIRTH study.

**Additional file 6.** Codebook Data Collectors, EN-BIRTH study.

**Additional file 7.** COREQ checklist, EN-BIRTH study.

**Additional file 8.** Care-to-documentation checklist, EN-BIRTH study.

**Additional file 9.** Labour and Delivery Registers, formal and informal, EN-BIRTH study.

**Additional file 10.** Labour ward routine register column design for maternal and newborn indicators, EN-BIRTH study.

**Additional file 11.** Demographic characteristics of respondents for barriers and enablers objective, labour/newborn wards, EN-BIRTH study.

**Additional file 12.** Labour ward care/documentation responsibilities by intervention, health worker respondents, EN-BIRTH study.

**Additional file 13.** Labour ward care/documentation responsibilities by intervention, research data collector respondents, EN-BIRTH study.

**Additional file 14.** Labour ward register order within all documentation, by indicator - health worker respondents, EN-BIRTH study.

**Additional file 15.** Labour ward register order within all documentation, by indicator - data collector respondents, EN-BIRTH study.

**Additional file 16.** Estimated minutes between care and documentation by indicator, care-documentation checklist, EN-BIRTH study.

**Additional file 17.** Ethical approval of local institutional review boards for EN-BIRTH study.

## Data Availability

The datasets generated during and/or analysed during the current study are available on LSHTM Data Compass repository, https://datacompass.lshtm.ac.uk/955/.

## Abbreviations

BD: Bangladesh
CEmONC: Comprehensive emergency obstetric and newborn care
CIFF: Children’s Investment Fund Foundation
DHS: The Demographic and Health Surveys Program
DHIS2: District Health Information Software 2
ENAP: *Every Newborn* Action Plan now branded as *Every Newborn*
EN-BIRTH: *Every Newborn*-Birth Indicators Research Tracking in Hospitals study
FGD: Focus Group Discussions
HIV: Human immunodeficiency virus
HMIS: Health Management Information Systems
icddr,b: International Centre for Diarrhoeal Disease Research, Bangladesh
IDI: In-depth interview
LMIC: low-and middle-income countries
MICS: Multiple Indicator Cluster Survey
NP: Nepal
PRISM: Performance of Routine Information System Management
TZ: Tanzania
COREQ: Consolidated criteria for reporting qualitative research
