# Supplementary material for: Barriers and enablers to routine register data collection for newborns and mothers: EN-BIRTH multi-country validation study
Source: BMC Pregnancy Childbirth. 2021 Mar 26;21(Suppl 1):233. doi: 10.1186/s12884-020-03517-3 (PMC7995573; doi:10.1186/s12884-020-03517-3)
Supplement: Supplementary file 1 — Additional file 1. National context and number of births in EN-BIRTH study hospital. [file 12884_2020_3517_MOESM1_ESM.pdf]

SUPPLEMENT TITLE:

Every Newborn BIRTH multi-country validation study: informing measurement of coverage and quality of maternal and newborn care

PAPER TITLE:

Barriers and enablers to routine register data collection for newborns and mothers: EN-BIRTH multi-country validation study

Additional File 1: National context and number of births in EN-BIRTH study hospital

**Country Context**	**Bangladesh**		**Nepal**	**Tanzania**	
**National mortality rates at start of EN-BIRTH study (2016)**					
MMR/ 100,000 live births (57)	176		258	398	
NMR/ 1000 live births (58)	21		22	22	
SBR/ 1000 total births (59)	25		18	22	
% Institutional Births (2016)(60)	47.1		57.4	62.6	
**EN-BIRTH Study Hospitals**					
**Name**	Maternal and Child Health Training Institute (MCHTI), Azimpur, Dhaka	Kushtia District Hospital	Pokhara Academy of Health Sciences	Temeke Regional Hospital, Dar es Salaam	Muhimbili National Hospital, Dar es Salaam
**Hospital type**	Tertiary	District	Regional	Regional	National
**Total births annual 2017-18**	3,346	2,887	9,422	11,609	8,233
