# Supplementary material for: Barriers and enablers to routine register data collection for newborns and mothers: EN-BIRTH multi-country validation study
Source: BMC Pregnancy Childbirth. 2021 Mar 26;21(Suppl 1):233. doi: 10.1186/s12884-020-03517-3 (PMC7995573; doi:10.1186/s12884-020-03517-3)
Supplement: Supplementary file 2 — Additional file 2. Summary of qualitative research methods to assess barriers and enablers to labour/newborn ward register documentation, EN-BIRTH study. [file 12884_2020_3517_MOESM2_ESM.pdf]

SUPPLEMENT TITLE:

Every Newborn BIRTH multi-country validation study: informing measurement of coverage and quality of maternal and newborn care

PAPER TITLE:

Barriers and enablers to routine register data collection for newborns and mothers: EN-BIRTH multi-country validation study

Additional File 2: Summary of qualitative research methods to assess barriers and enablers to labour/newborn ward register documentation, EN-BIRTH study

Method	Description of the method	Duty Ward	Responsibility	Selected indicator documented
**Heath workers:**				
**a) In-depth interviews and c)care-documentation checklist**	Nurses/midwives (n=4-6 per hospital, total n=30)	Labour and Delivery/ Operation Theatre	Care for patient and document	• Uterotonics to prevent PPH<br>• Early initiation of breastfeeding<br>• Neonatal bag mask ventilation
		Neonatal Ward/ Kangaroo Mother Care ward/corner	Care for patient and document	• Treatment of presumed infection<br>• Kangaroo Mother Care
	Doctors (n=2 per hospital, total n=10)	Labour and Delivery/ Operation Theatre/ Neonatal Ward/ Kangaroo Mother Care ward/corner	Care for patient and document	All indicators
**b) Focus Group Discussion**	Nurses-midwives from each ward (n=1 FGD per hospital, total respondents n=32)	Labour and Delivery/ Operation Theatre/ Neonatal Ward/ Kangaroo Mother Care ward/corner	Care for patient and document	All indicators
**EN-BIRTH data collectors:**				
**a) In-depth interviews and c) care-documentation checklist**	Data Trackers (n=3-4 per hospital, total n=19)	Registered patient at start of study	Observed care process and some content of documentation	Not applicable
	Clinical observers (n=4-8 per hospital, total n=24)	All wards	Observed care process but not content of documentation	All indicators
	Data Verifier/Extractor (n=1-4 per hospital, total n=13)	All wards	Extracted data from registers and patient notes for EN-BIRTH study	All indicators
	Supervisors (n=1-3 per hospital, total n=9)	All wards	Observed process and extracted data from registers and patient notes	All indicators
