# Supplementary material for: Barriers and enablers to routine register data collection for newborns and mothers: EN-BIRTH multi-country validation study
Source: BMC Pregnancy Childbirth. 2021 Mar 26;21(Suppl 1):233. doi: 10.1186/s12884-020-03517-3 (PMC7995573; doi:10.1186/s12884-020-03517-3)
Supplement: Supplementary file 3 — Additional file 3. Health Worker study guides in-depth interview (IDI) focus group discussion (FGD), EN-BIRTH study. [file 12884_2020_3517_MOESM3_ESM.pdf]

SUPPLEMENT TITLE:

Every Newborn BIRTH multi-country validation study: informing measurement of coverage and quality of maternal and newborn care

PAPER TITLE:

Barriers and enablers to routine register data collection for newborns and mothers: EN-BIRTH multi-country validation study

Additional File 3: Health Worker study guides in-depth interview (IDI) focus group discussion (FGD), EN-BIRTH study

Tool 3

(Doctors, Nurses, Midwives, Medical Assistants etc)

Instructions: For health workers who are working in Labour and Delivery ward, Operating Theatre and neonatal/ KMC ward. By health workers we mean Doctors, Nurses, Midwives, Medical assistants or other health Cadres.

Interviewer ID ___ ___ ___ ___ Interview date (*DD/MM/YYYY*): __ __ / __ __ / __ __ __ __

Hello, my name is __________________________ and I am from ______________________.
I would like to invite you to take part in an interview about documentation practices to collect health information for mothers and babies. Before you decide if you want to be interviewed, I would like to provide you with some information, please ask me any questions that come to mind.

London School of Hygiene & Tropical Medicine (LSHTM) in collaboration with Ifakara Health Institute (IHI) and Muhimbili University of Health and Allied Sciences (MUHAS) in Tanzania, International Centre for Diarrhoeal Disease Research, Bangladesh (icddr,b); UNICEF-Nepal with Lifeline in Nepal would like to learn about how the documentation of health information for mother and babies is done in this hospital and the barriers and enablers to routine recording and data usage. This is part of the Every Newborn - Birth Indicators Research Tracking in Hospitals (EN-BIRTH) study that is currently ongoing within this hospital. We will use the information to help understand and recommend improvements in the documentation practices for mothers and babies health care.

If you agree to participate, you will be interviewed by me at a time that is convenient to you. We will find a quiet place for the interview, which will take about an hour. I will write some notes and tape record the interview to help me remember all that was discussed. If you don't want me to take notes and tape record the interview, please let me know.

I will keep everything you say confidential, your name will not appear in any report and we will make sure that you cannot be identified. Taking part in the study may not benefit you directly, but may help us understand how to improve documentation in this hospital. Taking part in the study is voluntary. You can refuse to answer any question I ask or stop the interview at any time. You do not have to give a reason to refuse to take part or to stop the interview. Refusing to participate will not cause anything bad to happen. We do not pay people for being interviewed.

If you want to ask someone more about this research, the Principal Investigator contact is: Dr Honorati Masanja, Ifakara Health Institute (IHI), phone number - 022 277 4756, email: hmasanja@ihi.or.tz

If you want to ask anything about this research to someone else, who is independent of this project, please contact Dr. Mwifadhi Mrisho (Secretary of IHI ethical committee) at IHI P.O.BOX 78373 Dar-es-salaam, Phone number 022 277 4756, email: mmrisho@ihi.or.tz

I want to be sure you are taking part because you want to, so I am going to ask you to sign a form that says you agree to take part. If you do not want to participate that is OK, just let me know.

Now I would like to formally ask you to participate. If anything was unclear or you would like more information, please ask me. Do you agree to participate? [If the participant doesn't consent, thank the participant for listening].

Study participant consent:

I have read the information, or it has been read to me. I have had the opportunity to ask questions about it and any questions that I have asked have been answered to my satisfaction. I consent voluntarily to participate as a participant in this study.

By putting my signature below, I certify that I have read/listened and understood the information and agree to participate in the study.

Name of Participant: ____

Signature of the Participant: ___________________________________Date: _________________________

Name of the Interviewer: ____

Signature of the Interviewer: ___________________________________Date: ___________________________

DO NOT proceed without their consent.

Interviewer ID ___ ___ ___ ___ Interview date (*DD/MM/YYYY*): __ __ / __ __ / __ __ __

Location of the interview___________________________________
Country: ____
Name of facility: _______________________________________

Introduction: Ensure that the participant is comfortable, has time to participate in the interview.

Explain you are starting the audio recorder.

Time interview started: : ☐☐ ☐☐

THEME 1: Socio-demographic information of the respondents

**First Name**	**Last name**	**Designation**	**Years worked as health workers**	**Highest Education (in grade and degree)**	**Age**	**Gender**

How many years/months have you worked in these different wards?

	Labour & Delivery	Operating Theatre	KMC ward	Neonatal ward
**Number of years/ months**				
**Questions to answer**	**1.1 and 2 to 15**	**1.2 and 2 to 15**	**1.3 and 2 to 15**	**1.4 and 2 to 15**

Explain again to the participant that:

- *I want to learn about your experience, thoughts and perspective on this topic of routine documentation in facility registers and other record books*
- *There is NO 'right' or 'wrong' answer. If you don't know about a question that I ask you, please tell me.*
- *The themes that emerge in your interview will be put together with other interviews including groups of health workers, data collectors who worked on the research study and management interviews to help get a wide understanding of what people think about this topic.*
- *I will explain our definitions: By documents and documentation we mean – recording of information about mothers and babies e.g: L&D Register, KMC Register, OT Register, Neonatal ward and other record books (unofficial/official or informal/ formal), patient records and monthly summary sheets/books etc.*

[Theme 2: INPUT – Behavioural, Technical, Organizational factors]

1 Documentation by location

1.1 In Labour and Delivery ward in this facility [Ask Health Workers who currently work in Labour & Delivery (L&D)]

1.1.1 Who does the documentation of routine information for mothers and babies in your work place in L&D?
Probes: Who helps them? Where do they write? What documents/ registers do they fill-up? Is it all on paper or on computer or both?

1.1.2 Can you describe how you feel about documentation of routine information for mothers and babies in your work place in L&D?

1.1.3 There are different types of documents in L&D (e.g. clinical registers, patient case notes, patient held records, monthly summary sheets, others?) Why do we have all these different types? What are they used for?
Probes: - Sometimes there are formal documents and some are informal (counter books/ hand written books)? Why are there both?

1.1.4 Information (variables) record in the different documents – which are they the same or different?
Probes: Is everything you need to document in the register also found in the patient notes? Any variables missing? Do they overlap? Do they align? What information is typically missing?

1.1.5 What is your opinion about current documentation process and flow? Do you have what you need for documentation?
Probes: Any relation to resources/logistics e.g. registers/pen/papers/copies of partogragh?
How do you feel about the design of the registers/ patient records/ files? Is it helpful for you? Is it clear to see when information is incomplete? Is the documentation process and flow good or not? Do the documents change? How often? Have the documents (registers, patient case records) or the documentation process changed in the last year? What are you struggling with and what helps you?

1.1.6 How do you feel about the relationship of caring for the patient AND documenting your care? Which is important?
Probes: Why? Does documentation help care or hinder (obstruct) care?

1.1.7 When do you document in relation to the care of patient?
Probes: When do you write? Relationship between care and documentation i.e. during provision of care, how you actually do both tasks of caring for the patient AND documentation – do you do together or one before the other? If documented later, how long after you give care?

1.1.8 How long does documenting usually take you? How much of your time does it take? If you divide up how much of your day is spent in documenting
Probes: Try to get an estimate time e.g. number of hours or proportion of duty e.g. half of your day?

1.1.9 Are some pieces of information (data points, data elements, indicators) recorded better than others? Can you explain why this might be?
Probes: give examples of data according to that ward eg Birth Weight, feeding practice for KMC babies

1.1.10 Can you always document care immediately after it is given or sometimes does some time elapse between the care and the documentation?
Probe: How long for what information? What causes the delay? How do you remember the information before you write it down? What factors help or hinder the timing of documentation

1.1.11 Of the different documents that are filled in – which do you think are less or more important? I will list the documents and ask you to tell me which are the most important for 4 groups: health workers, patient, hospital management and Health Management Information System (HMIS)?
0 = not important 1 = less important 2 = important, 3 = more/very important, 4 = don't know
*Instructions – number 1 through 3 in each column. Use this table to help stimulate discussion of the respondent's perception comparing the importance using documentation for quality of patient care **versus** finances **versus** reporting etc?*

	Document name	For the Health care workers?	For the patient?	For hospital management?	For HMIS?
a) Registers clinical	L&D register				
b) Patient case notes (stay in facility)					
c) Patient held records					
d) Monthly Summary sheets					
e) Other	e.g. Insurance				
f) Other	e.g. Research				
g) Other	e.g Computer				
h) Other	e.g.				
i) Other	e.g.				
j) Other	e.g.				

1.1.12 For these documents we have been talking about, what was your reasoning as to why they are more or less important? *Capture some of the reasons they gave to fill the table*

1.1.13 We are interested about some specific intervention documentation - where they are documented (put a tick) and if you have any comments about the documentation of these variables?

	a)	b)	c)	d)	e)	Comments
	Registers clinical = L&D	Patient case notes	Patient held records e.g. postnatal card	Monthly Summary sheets	other =	
1.Uterotonic (prophylactic)						
2.First time breast feeding						
3.Baby Resuscitation						
4.Management of neonatal infection						
5.KMC						
6.Antenatal Corticosteroids						

1.1.14 Can you describe any challenges or difficulties you have experienced in documentation in L&D?
Probes: Are there many registers or papers to fill? Do you find the documentation complicated to complete or not? Does it take a long time? Are the things you need all in one place? Do you think it is well organized? Do you write anywhere else (e.g. small piece of paper/ notebook)?

1.2 In Operating Theatre in this facility [Ask Health Workers who currently work in Operating Theatre (OT)]

1.2.1 Who does the documentation of routine information for mothers and babies in your work place in OT?
Probes: Who helps them? Where do they write? What documents/ registers do they fill-up? Is it all on paper or on computer or both?

1.2.2 Can you describe how you feel about documentation of routine information for mothers and babies in your work place in OT?

1.2.3 There are different types of documents in OT (e.g. clinical registers, patient case notes, patient held records, monthly summary sheets, others?) Why do we have all these different types? What are they used for?
Probes: - Sometimes there are formal documents and some are informal (counter books/ hand written books)? Why are there both?

1.2.4 Information (variables) record in the different documents – where are they the same or different?
Probes: Is everything you need to document in the register also found in the patient notes? Any variables missing? Do they overlap? Do they align? What information is typically missing?

1.2.5 What is your opinion about current documentation process and flow? Do you have what you need for documentation?
Probes: Any relation to resources/logistics e.g. registers/pen/papers/copies of partogragh?
How do you feel about the design of the registers patient records/ files? Is it helpful for you? Is it clear to see when information is incomplete? Is the documentation process and flow good or not? Do the documents change? How often? Have they changed in the last year? What are you struggling with and what helps you?

1.2.6 How do you feel about the relationship of caring for the patient AND documenting that care? Which is important?
Probes: does documentation help care or hinder care?

1.2.7 When do you document in relation to the care?
Probes: When do you write? Relationship between care and documentation i.e. during provision of care, how you actually do both tasks of caring for the client AND documentation – do you do together or one before the other? If documented later, how long after you give care?

1.2.8 How long does documenting usually take you? How much of your time does it take? If you divide up how much of your day is spent documenting
Probes: Try to get an estimate eg. half of your day?

1.2.9 Are some pieces of information (data points, data elements, indicators) recorded better than others? Can you explain why this might be?

1.2.10 Can you always document care immediately after it is given or sometimes does some time elapse between the care and the documentation?
Probe: How long for what information? What causes the delay? How do you remember the information before you write it down?

1.2.11 Of the different documents that are filled in – which do you think are less or more important? I will list the documents and ask you to tell me which are the most important for 4 groups: health workers, patient, hospital management and Health Management Information System (HMIS)?
0 = not important 1 = less important 2 = important, 3 = more/very important
Instructions – number 1 through 3 in each column. Use this table to help stimulate discussion of the respondent's perception comparing the importance using documentation for quality of patient care ***versus*** *finances* ***versus*** *reporting etc?*

	Name of documents	For the Health care workers?	For the patient?	For hospital management?	For the HMIS?
a) Registers clinical	L&D register				
b) Patient case notes (stay in facility)					
c) Patient held records					
d) Monthly Summary sheets					
e) Other	e.g. Insurance				
f) Other	e.g. Research				
g) Other	e.g Computer				
h) Other	e.g.				
i) Other	e.g.				
j) Other	e.g.				

1.2.12 For these documents we have been talking about, what was your reasoning as to why they are more or less important? *Capture some of the reasons they gave to fill the table*

1.2.13 We are interested about some specific intervention documentation - where they are documented (put a tick) and if you have any comments about the documentation of these variables?

	a)	b)	c)	d)	e)	Comments
	Registers clinical = L&D	Patient case notes	Patient held records e.g. postnatal card	Monthly Summary sheets	other =	
1.Uterotonic (prophylactic)						
2.First time breast feeding						
3.Baby Resuscitation						
4.Management of neonatal infection						
5.KMC						
6.Antenatal Corticosteroids						

1.2.14 Can you describe any challenges or difficulties you have experienced in documentation in OT?
Probes: Are there many registers or papers to fill? Do you find the documentation complicated to complete or not? Does it take a long time? Are the things you need all in one place? Do you think it is well organized? Do you write anywhere else (e.g. small piece of paper/ notebook)?

1.3 In KMC ward in this facility [Ask Health Workers who currently work in KMC ward]

1.3.1 Who does the documentation of routine information for mothers and babies in your work place in KMC ward?
Probes: Who helps them? Where do they write? What documents/ registers do they fill-up? Is it all on paper or on computer or both?

1.3.2 Can you describe how you feel about documentation of routine information for mothers and babies in your work place in KMC ward?

1.3.3 There are different types of documents in KMC ward (e.g. clinical registers, patient case notes, patient held records, monthly summary sheets, others?) Why do we have all these different types? What are they used for?
Probes: - Sometimes there are formal documents and some are informal (counter books/ hand written books)? Why are there both?

1.3.4 Information (variables) record in the different documents – where are they the same or different?
Probes: Is everything you need to document in the register also found in the patient notes? Any variables missing? Do they overlap? Do they align? What information is typically missing?

1.3.5 What is your opinion about current documentation process and flow? Do you have what you need for documentation?
Probes: Any relation to resources/logistics e.g. registers/pen/papers/copies of partogragh?
How do you feel about the design of the registers patient records/ files? Is it helpful for you? Is it clear to see when information is incomplete? Is the documentation process and flow good or not? Do the documents change? How often? Have they changed in the last year? What are you struggling with and what helps you?

1.3.6 How do you feel about the relationship of caring for the patient AND documenting that care? Which is important?
Probes: does documentation help care or hinder (obstruct) care?

1.3.7 When do you document in relation to the care?
Probes: When do you write? Relationship between care and documentation i.e. during provision of care, how you actually do both tasks of caring for the client AND documentation – do you do together or one before the other? If documented later, how long after you give care?

1.3.8 How long does documenting usually take you? How much of your time does it take? If you divide up how much of your day is spent documenting
Probes: Try to get an estimate eg. half of your day?

1.3.9 Are some pieces of information (data points, data elements, indicators) recorded better than others? Can you explain why this might be?

1.3.10 Can you always document care immediately after it is given or sometimes does some time elapse between the care and the documentation?
Probe: How long for what information? What causes the delay? How do you remember the information before you write it down?

1.3.11 Of the different documents that are filled in – which do you think are less or more important? I will list the documents and ask you to tell me which are the most important for 4 groups: health workers, patient, hospital management and Health Management Information System (HMIS)?
0 = not important 1 = less important 2 = important, 3 = more/very important
Instructions – number 1 through 3 in each column. Use this table to help stimulate discussion of the respondent's perception comparing the importance using documentation for quality of patient care ***versus*** *finances* ***versus*** *reporting etc?*

	Name of documents	For Health care workers?	For the patient?	For hospital management?	For the HMIS?
a) Registers clinical	KMC register				
b) Patient case notes (stay in facility)					
c) Patient held records					
d) Monthly Summary sheets					
e) Other	e.g. Insurance				
f) Other	e.g. Research				
g) Other	e.g. computer				
h) Other					
i) Other					
j) Other					

1.3.12 We are interested about some specific intervention documentation - where they are documented (put a tick) and if you have any comments about the documentation of these variables?

	a)	b)	c)	d)	e)	Comments
	Registers clinical = L&D	Patient case notes	Patient held records	Monthly Summary sheets	other =	
1.Uterotonic (prophylactic)	NOT APPLICABLE					
2.First time breast feeding						
3.Baby Resuscitation						
4.Management of neonatal infection						
5.KMC						
6.Antenatal Corticosteroids						

1.3.13 Can you describe any challenges or difficulties you have experienced in documentation in KMC ward?
Probes: Are there many registers or papers to fill? Do you find the documentation complicated to complete or not? Does it take a long time? Are the things you need all in one place? Do you think it is well organized? Do you write anywhere else (e.g. small piece of paper/ notebook)?

1.4 In Neonatal ward in this facility [Ask Health Workers who currently work in Neonatal ward]

1.4.1 Who does the documentation of routine information for mothers and babies in your work place in Neonatal Ward?
Probes: Who helps them? Where do they write? What documents/ registers do they fill-up? Is it all on paper or on computer or both?

1.4.2 Can you describe how you feel about documentation of routine information for mothers and babies in your work place in Neonatal Ward?

1.4.3 There are different types of documents in Neonatal Ward (e.g. clinical registers, patient case notes, patient held records, monthly summary sheets, others?) Why do we have all these different types? What are they used for?
Probes: - Sometimes there are formal documents and some are informal (counter books/ hand written books)? Why are there both?

1.4.4 Information (variables) record in the different documents – where are they the same or different?
Probes: Is everything you need to document in the register also found in the patient notes? Any variables missing? Do they overlap? Do they align? What information is typically missing?

1.4.5 What is your opinion about current documentation process and flow? Do you have what you need for documentation?
Probes: Any relation to resources/logistics e.g. registers/pen/papers/copies of partogragh?
How do you feel about the design of the registers patient records/ files? Is it helpful for you? Is it clear to see when information is incomplete? Is the documentation process and flow good or not? Do the documents change? How often? Have they changed in the last year? What are you struggling with and what helps you?

1.4.6 How do you feel about the relationship of caring for the patient AND documenting that care? Which is important?
Probes: does documentation help care or hinder (obstruct) care?

1.4.7 When do you document in relation to the care?
Probes: When do you write? Relationship between care and documentation i.e. during provision of care, how you actually do both tasks of caring for the client AND documentation – do you do together or one before the other? If documented later, how long after you give care?

1.4.8 How long does documenting usually take you? How much of your time does it take? If you divide up how much of your day is spent documenting
Probes: Try to get an estimate eg. half of your day?

1.4.9 Are some pieces of information (data points, data elements, indicators) recorded better than others? Can you explain why this might be?

1.4.10 Can you always document care immediately after it is given or sometimes does some time elapse between the care and the documentation?
Probe: How long for what information? What causes the delay? How do you remember the information before you write it down?

1.4.11 Of the different documents that are filled in – which do you think are less or more important? I will list the documents and ask you to tell me which are the most important for 4 groups: health workers, patient, hospital management and Health Management Information System (HMIS)?
0 = not important 1 = less important 2 = important, 3 = more/very important
*Instructions – number 1 through 3 in each column. Use this table to help stimulate discussion of the respondent's perception comparing the importance using documentation for quality of patient care **versus** finances **versus** reporting etc?*

	Name of documents	For Health care workers?	For the patient?	For hospital management?	For the HMIS?
a) Registers clinical	L&D register				
b) Patient case notes (stay in facility)					
c) Patient held records					
d) Monthly Summary sheets					
e) Other	Insurance				
f) Other	Research				
g) Other	computer				
h) Other					
i) Other					
j) Other					

1.4.12 We are interested about some specific intervention documentation - where they are documented (put a tick) and if you have any comments about the documentation of these variables? (*note 1. Uterotonic removed)*

	a)	b)	c)	d)	e)	Comments
	Registers clinical = L&D	Patient case notes	Patient held records	Monthly Summary sheets	other =	
1.Uterotonic (prophylactic)	NOT APPLICABLE					
2.First time breast feeding						
3.Baby Resuscitation						
4.Management of neonatal infection						
5.KMC						
6.Antenatal Corticosteroids						

1.4.13 Can you describe any challenges or difficulties you have experienced in documentation in Neonatal Ward?
Probes: Are there many registers or papers to fill? Do you find the documentation complicated to complete or not? Does it take a long time? Are the things you need all in one place? Do you think it is well organized? Do you write anywhere else (e.g. small piece of paper/ notebook)?

[Ask questions 2 to 19 to all respondents]

[INPUT - Behavioural Factors]

[Motivation]

2 Why do you think anything is documented in this facility - in registers? In patient records?
Probe: What motivators are there for this documentation to be done?

3 Where does the initiative come from to do this documentation?
Probes: From Health Workers? Supervisors (ward-in-charge)? Superiors (hospital managers, directors)? Outside demand (other NGO/government/UN agency eg WHO as appropriate)? Patients and their families?

4 Can you describe the importance of documentation is in this facility?
Probes: Do you think some information is given more importance or more attention in documentation than other information? Please give examples of documentation that seem to be more important or less important or not important. Why do you think this is the case? Is the information used in different ways? Is the documentation and information important to the health workers? Important to Supervisors? Important to people outside the facility? Is it considered as only a task that needs to be done?

5 What is your perspective about the "culture of information and data" in this facility?
Probe: is information and data valued in this facility? How is data valued? Is an enabling environment for data recording generated or supported by unit support or hospital management / Director? Any prize/award/recognition?

[INPUT - Organisational Factors]

[Training & supervision]

6 Have you ever had any on-the-job training or supervision about documentation?
Probes: What training did you have? How it was done? Where was it done? Who was facilitating? Who was it done for? Was it relevant to the implementation on the ward? How often did you see? What was the atmosphere like – blaming? Criticising? Supportive? Do you feel you have been trained appropriately, you feel confident? any concerns?

[Quality]

7 Can you describe any situation when you have noticed any issues with your own documentation quality?
Probes: discrepancies between register and patient record? Missing information? Which parts of routine documentation are completed less well or incomplete and why? Handwriting difficult to read? eg. Yes= "N" and No="H" in Tanzania can be difficult to read handwriting.

[Availability of resources]

8 Can you describe what you have seen about the availability of the documents for mother and baby information?
Probe: L&D Register, KMC Register, OT Register, Neonatal ward and other record books (unofficial/official or informal/ formal), patient records and monthly summary sheets/books – are they always available? Sometimes available? Can you give example of any shortage or stock-outs? If yes, how did they solve? Who solved it?

9 Can you describe other resources needed for documentation and their availability?
Probe: Examples: pens, people. Always available? Sometimes available? Can you give an example of any shortage or stock-outs? If yes, how did they solve? Who solved it?

[THEME 3: PROCESS - RHIS]

10 Have you ever seen any data quality check about your documentation?
Probes: if yes, can you describe what did you see? How it was done? Where it was done? Who did it? How often was it checked? How did you feel? What was the atmosphere like – blaming? Criticising? Supportive?

11 When patients are moved between clinical areas, how does this affect documentation and recording?
Example: From Antenatal ward to L&D, from L&D to OT, from OT to recovery, from L&D or OT to postnatal/neonatal/KMC ward. From inpatient care to discharge? Is there any delay in availability of information?

Probes: Do health workers hand information over verbally or written? Do the documents always stay with the patients or sometimes the patient and the notes get separated? For example: mother in OT and nurse takes partograph back to L&D to use it to write in register? Does a patient who delivers in OT have their information written in L&D register (e.g. is that L&D in OT or other location? Or baby goes to neonatal ward but the papers stay with on the ward where the mother is? How is information transported within the facility? Any problems? Any suggestions to improve?

12 How is information transmitted from the facility up the health system – eg to the district, national level?
Probes: Who manages/processes the information in your facility? How is information transmitted from the facility outside? How often? How do you feel about the deadlines to submit this information - is there ever any delay in transmitting this information? Any problems? Any suggestions to improve?

13 Do you ever get any feedback from the data that you send up the health system?
Probes: What information do you receive from the HMIS? What reports? How often? Who provides the feedback? How is the feedback provided? How did you feel? What was the atmosphere like – blaming? Criticising? Supportive? Any problems? Any suggestions to improve?

[THEME 4: OUTPUT - Information use]

14 Can you describe how this documentation is used by anyone?
Probe: Who uses the data? What information is used? What is it used for? How often is it used? Are there some parts that are used more than others? How do you use the information?

[THEME 5: OUTCOMES - Opportunities and recommendations]

15 In your opinion – for the recording and documentation of maternal and newborn health information how could it be improved at this facility?
Probe: training, logistics, management, supervision, monitoring, capacity development, reporting, resources etc.

16 What do you think the barriers and constraints to this improvement could be?

17 How do you feel about your role in the documentation and information about mothers and babies in your facility?
Probe: Is the information you collect enough?

18 What is your opinion to use the compiled data for mothers and babies on the wider HMIS at district/national level?
Probe: What is the role of the HMIS platform? How important is it? Does the right information go into the HMIS system from your facility? Any suggestions? Do all health workers know about the HMIS data transmission?

19 Is there anything else you would like to add about your experiences with, or views on, any of these documentation and recording processes?

Time interview end: ☐☐:☐☐

Thank the participant for his/her time. Remind them that the information will be kept confidential.

Interviewer comments on how the interview went:

e.g. Optional - describe how the interview went. Any distractions etc. Make optional brief notes
