# Supplementary material for: Barriers and enablers to routine register data collection for newborns and mothers: EN-BIRTH multi-country validation study
Source: BMC Pregnancy Childbirth. 2021 Mar 26;21(Suppl 1):233. doi: 10.1186/s12884-020-03517-3 (PMC7995573; doi:10.1186/s12884-020-03517-3)
Supplement: Supplementary file 4 — Additional file 4. Data Collector study guides in-depth interview (IDI), EN-BIRTH study. [file 12884_2020_3517_MOESM4_ESM.pdf]

SUPPLEMENT TITLE:

Every Newborn BIRTH multi-country validation study: informing measurement of coverage and quality of maternal and newborn care

PAPER TITLE:

Barriers and enablers to routine register data collection for newborns and mothers: EN-BIRTH multi-country validation study

Additional File 4: Data Collector study guides in-depth interview (IDI), EN-BIRTH study

Trackers, Clinical Observers, Data Verifiers and Extractors and Supervisors

Instructions: Clinical Observers (CO) who are observing deliveries in Labour and Delivery ward and Theatre and babies receiving KMC.

Interviewer ID ___ ___ ___ ___   Interview date (*DD/MM/YYYY*): __ __ / __ __ / __ __ __ __

Hello, my name is __________________________ and I am from _______________________.
I would like to invite you to take part in an interview about documentation activities for mothers and babies health information. Before you decide if you want to be interviewed, I would like to provide you with some information, please ask me any questions that come to mind.

London School of Hygiene & Tropical Medicine and LSHTM in collaboration with Ifakara Health Institute (IHI) and Muhimbili University of Health and Allied Sciences (MUHAS) in Tanzania, International Centre for Diarrhoeal Disease Research, Bangladesh (icddr,b); UNICEF-Nepal with Lifeline in Nepal would like to learn about how the documentation of health information for mother and babies is done in this hospital and the barriers and enablers to routine recording and data usage. This is part of the "The Every Newborn - Birth Indicators Research Tracking in Hospitals (EN-BIRTH) study that is currently ongoing within this hospital. We will use the information to help to understand and recommend improvements in the documentation activities for mothers and babies health information.

If you agree to participate, you will be interviewed by me at a time that is convenient to you. We will find a quiet place for the interview, which will take about an hour. I will write some notes and tape record the interview to help me remember all that was discussed. If you don't want me to take notes and tape record the interview, please let me know.

I will keep everything you say confidential, your name will not appear in any report and we will make sure that you cannot be identified. Taking part in the study may not benefit you directly, but may help us understand how to improve documentation in this hospital. Taking part in the study is voluntary. You can refuse to answer any question I ask or stop the interview at any time. You do not have to give a reason to refuse to take part or to stop the interview. Refusing to participate will not cause anything bad to happen. We do not pay people for being interviewed.

If you want to ask someone more about this research, the Principal Investigator contact is: Dr. Godfrey Mbaruku, Ifakara Health Institute (IHI), Phone number - 0784 492 129, email: gmbaruku@ihi.or.tz

If you want to ask anything about this research to someone else, who is independent of this project, please contact Dr. Mwifadhi Mrisho (Secretary of IHI ethical committee) at IHI P.O.BOX 78373 Dar-es-salaam, Phone number 022 277 4756, email: mmrisho@ihi.or.tz

Study participant consent:
I want to be sure you are taking part because you want to, so I am going to ask you to sign a form that says you agree to take part. If you do not want to participate that is OK, just let me know.

Now I would like to formally ask you to participate. If anything was unclear or you would like more information, please ask me. Do you agree to participate? [If the participant doesn't consent; Thank the participant for listening].

I have read the information, or it has been read to me. I have had the opportunity to ask questions about it and any questions that I have asked have been answered to my satisfaction. I consent voluntarily to participate as a participant in this study.

By putting my signature below, I certify that I have read/listened and understood the information and agree to participate in the study.

Name of Participant: ____

Signature of the Participant: ____________________________________Date: _________________________

Name of the Interviewer: ____

Signature of the Interviewer: ____________________________________Date: _________________________

DO NOT proceed without their consent.

Interviewer ID ___ ___ ___ ___ Interview date (*DD/MM/YYYY*): __ __ / __ __ / __ __ __

Location of the interview__________________________________
Country: ____
Name of facility: ______________________________________

Introduction: Ensure that the participant is comfortable, has time to participate in the interview.

Explain you are starting the audio recorder.

Time interview started: ☐☐:☐☐

Theme 1: Socio-demographic information of the respondents

**First Name**	**Last name**	**Highest Education**	**Age**	**Gender**

How many months have you worked in these areas?

	Trackers	Clinical Observers in Labour & Delivery	Clinical Observers in Operating Theatre	Clinical Observers in KMC ward	Clinical Observers in Neonatal ward	Data Verifier & Extractor	Supervisor
**Write number of months**							
**Questions to answer**	**1.1 and 2 to 15**	**1.2 and 2 to 15**	**1.3 and 2 to 15**	**1.4 and 2 to 15**	**1.5 and 2 to 15**	**1.2, 1.3, 1.4, 1.5 and 2 to 15**	**All questions**

Explain again to the participant that:

- *I want to learn about your experience, thoughts and perspective on this topic of routine documentation in facility registers and other record books*
- *There is NO 'right' or 'wrong' answer.*
- *The themes that emerge in your interview will be put together with other data collector, health worker and management interviews including groups to help get a wide understanding of what people think about this topic.*
- *I will explain our definitions: By documents and documentation we mean – recording of information about mothers and babies in for example: L&D Register, KMC Register, OT Register, Neonatal ward and Antenatal ward Register and other record books (unofficial and official) and patient records and monthly summary books etc*

[Theme 2: INPUT – Behavioural, Technical, Organizational factors]

1 <u>Documentation by location</u>

1.1 <u>At the place of routine admission to this facility</u>
<u>[Ask Trackers]</u>

1.1.1 Can you describe how routine information for mothers and babies is typically documented at admission?
Probes: who does it? Who helps them? Where do they write? What documents/ registers do they fill-up? Do you see them write anywhere else (e.g. small piece of paper)? Is it all on paper or on computer or both?

1.1.2 Please describe the typical documentation process (order of events) that you have seen at admission.
Probes: When do they write? Relationship between care and documentation i.e. during provision of care, how does the person actually do both tasks of caring for the client AND documentation – do they do together or one before the other? If documented later, how long after the care is given?

1.1.3 Why do you think they do it this way at admission?
Probes: what are their other roles / responsibilities? Any relation to resources/logistics e.g. registers/pen/papers/copies of partogragh*? Where are the documents or registers situated – in one place? On different tables? What is the physical distance between the documents and the patient?*

1.1.4 What is your opinion about their current documentation process and flow?
Probe: is it a good flow or not? Why do you think so?

1.1.5 Can you describe any challenges or difficulties or barriers you have observed in documentation at admission?
Probes: Are there many registers or papers to fill? Do you think health workers find the documentation complicated to complete or not? Does it take a long time? Are the things they need all in one place? Do you think it is well organized?

1.1.6 We are not asking you specifically about *what* is written in the routine facility registers /documents, but we are interested if you observed anything about some specific intervention documentation - *Who* documented? *How* did they document? *When* did they document? The interventions of interest are:

1.1.6.1 Antenatal Corticosteroids?

1.2 <u>In Labour and Delivery (L&D) ward in this facility</u>
[Ask Clinical Observers in Labour & Delivery AND Data Verifier & Extractor]

1.2.1 Can you describe how routine information for mothers and babies is typically documented in L&D?
Probes: who does it? Who helps them? Where do they write? What documents/ registers do they fill-up? Do you see them write anywhere else (e.g. small piece of paper)? Is it all on paper or on computer or both?

1.2.2 Please describe the typical documentation process (order of events) that you have seen in L&D.
Probes: When do they write? Relationship between care and documentation i.e. during provision of care, how does the person actually do both tasks of caring for the client AND documentation – do they do together or one before the other? If documented later, how long after the care is given?

There are many examples of documentation, and I will give you just one example now: After a baby is born, they are weighed. Can you describe the process of taking the baby, weighing the baby and writing the weight in relevant documents? Exactly who does what? Where do they weigh? Where document? When record? Can you describe the order of events for documentation of BW?

1.2.3 *Can you describe order of events for other examples of documentation that you have seen in L&D?*
Why do you think they do it this way in L&D?
Probes: what are their other roles / responsibilities? Any relation to resources/logistics e.g. registers/pen/papers/copies of partogragh*? Where are the documents or registers situated – in one place? On different tables? What is the physical distance between the documents and the patient?*

1.2.4 What is your opinion about their current documentation process and flow?
Probe: is it a good flow or not?

1.2.5 Can you describe any challenges or difficulties or barriers you have observed in documentation in L&D?
Probes: Are there many registers or papers to fill? Do you think health workers find the documentation complicated to complete or not? Does it take a long time? Are the things they need all in one place? Do you think it is well organized?

1.2.6 We are not asking you specifically about *what* is written in the routine facility registers /documents, but we are interested if you observed anything about some specific intervention documentation - *Who* documented? *How* did they document? *When* did they document? The interventions of interest are:

1.2.6.1 Uterotonic (prophylactic)?
1.2.6.2 Essential Newborn Care (first time breast feeding)?
1.2.6.3 Resuscitation of the baby?
1.2.6.4 Antenatal Corticosteroids?

1.3 <u>In Operating Theatre (OT) in this facility</u>

[Ask Clinical Observers in Operating Theatre AND Data Verifier & Extractor]

1.3.1 Can you describe how routine information for mothers and babies is typically documented in OT?
Probes: who does it? Who helps them? Where do they write? What documents/ registers do they fill-up? Do you seem them write anywhere else (e.g. small piece of paper)? Is it all on paper or on computer or both?

1.3.2 Please describe the typical documentation process (order of events) that you have seen in OT?
Probes: When do they write? Relationship between care and documentation i.e. during provision of care, how does the person actually do both tasks of caring for the client AND documentation – do they do together or one before the other? If documented later, how long after the care is given?

There are many examples of documentation, and I will give you just one example now: After a baby is born, they are weighed. Can you describe the process of taking the baby, weighing the baby and writing the weight in relevant documents? Exactly who does what? Where do they weigh? Where document? When document? Can you describe the order of events for documentation of BW?

Can you describe order of events for other examples of documentation that you have seen in OT?

1.3.3 Why do you think they do it this way in OT?
Probes: what are their other roles / responsibilities? Any relation to resources/logistics e.g. registers/pen/papers/copies of partogragh*? Where are the documents or registers situated – in one place? On different tables? What is the physical distance between the documents and the patient?*

1.3.4 What is your opinion about their current documentation process and flow?
Probe: is it a good flow or not?

1.3.5 Can you describe any challenges or difficulties or barriers you have observed in documentation in OT?
Probes: Are there many registers or papers to fill? Do you think health workers find the documentation complicated to complete or not? Does it take a long time? Are the things they need all in one place? Do you think it is well organized?

1.3.6 We are not asking you specifically about *what* is written in the routine facility registers /documents, but we are interested if you observed anything about some specific intervention documentation - *Who* documented? *How* did they document? *When* did they document? The interventions of interest are:

1.3.6.1 Uterotonic (prophylactic)?
1.3.6.2 Essential Newborn Care (1st breast feed)?
1.3.6.3 Resuscitation of the baby?
1.3.6.4 Antenatal Corticosteroids?

1.4 <u>In KMC ward in this facility</u>

[Ask Clinical Observers in KMC ward AND Data Verifier & Extractor]

1.4.1 Can you describe how routine information for mothers and babies is typically documented in KMC ward?
Probes: who does it? Who helps them? Where do they write? What documents/ registers do they fill-up? Do you see them write anywhere else (e.g. small piece of paper)? Is it all on paper or on computer or both?

1.4.2 Please describe the typical documentation process (order of events) that you have seen in KMC ward.
Probes: When do they write? Relationship between care and documentation i.e. during provision of care, how does the person actually do both tasks of caring for the client AND documentation – do they do together or one before the other? If documented later, how long after the care is given?

There are many examples of documentation, and I will give you just one example now: When a baby is put in KMC. Describe the process of taking the baby, positioning the baby in KMC and writing the KMC care in relevant documents. Exactly who does what? Where do they do the KMC? Where do they document? When do they document? Can you describe the order of events for documentation of KMC?

Can you describe order of events for other examples of documentation that you have seen in KMC ward?

1.4.3 Why do you think they do it this way in KMC ward?
Probes: what are their other roles / responsibilities? Any relation to resources/logistics e.g. registers/pen/papers/copies of partogragh*? Where are the documents or registers situated – in one place? On different tables? What is the physical distance between the documents and the patient?*

1.4.4 What is your opinion about their current documentation process and flow?
Probe: is it a good flow or not?

1.4.5 Can you describe any challenges or difficulties or barriers you have observed in documentation in KMC?
Probes: Are there many registers or papers to fill? Do you think health workers find the documentation complicated to complete or not? Does it take a long time? Are the things they need all in one place? Do you think it is well organized?

1.4.6 We are not asking you specifically about *what* is written in the routine facility registers /documents, but we are interested if you observed anything about some specific intervention documentation - *Who* documented? *How* did they document? *When* did they document? The interventions of interest are:

1.4.6.1 Management of neonatal infection?

1.5 In Neonatal ward in this facility
[Ask Clinical Observers in Neonatal ward AND Data Verifier & Extractor]

1.5.1 Can you describe how routine information for mothers and babies is typically documented in neonatal ward?
Probes: who does it? Who helps them? Where do they write? What documents/ registers do they fill-up? Do you see them write anywhere else (e.g. small piece of paper)? Is it all on paper or on computer or both?

1.5.2 Please describe the typical documentation process (order of events) that you have seen in neonatal ward.
Probes: When do they write? Relationship between care and documentation i.e. during provision of care, how does the person actually do both tasks of caring for the client AND documentation – do they do together or one before the other? If documented later, how long after the care is given?

1.5.3 Why do you think they do it this way in neonatal ward?
Probes: what are their other roles / responsibilities? Any relation to resources/logistics e.g. registers/pen/papers/copies of partogragh*? Where are the documents or registers situated – in one place? On different tables? What is the physical distance between the documents and the patient?*

1.5.4 What is your opinion about their current documentation process and flow?
Probe: is it a good flow or not?

1.5.5 Can you describe any challenges or difficulties or barriers you have observed in documentation in neonatal ward?
Probes: Are there many registers or papers to fill? Do you think health workers find the documentation complicated to complete or not? Does it take a long time? Are the things they need all in one place? Do you think it is well organized?

1.5.6 We are not asking you specifically about *what* is written in the routine facility registers /documents, but we are interested if you observed anything about some specific intervention documentation - *Who* documented? *How* did they document? *When* did they document? The interventions of interest are:

1.5.6.1 Management of neonatal infection?

[Ask questions 2 to 15 to all respondents]

2 When patients are moved between clinical areas, have you seen any effect on documentation and recording?

Example: From Antenatal ward to L&D, from L&D to OT, from OT to recovery, then to postnatal ward and discharge.

Probes: Do health workers hand information over verbally or written? Do the documents always stay with the patients or sometimes the patient and the notes get separated? For example: mother in OT and nurse takes partograph back to L&D to use it to write in register? Or baby goes to neonatal ward but the papers stay with the mother?

[Motivation]

3 Why do you think anything is documented in this facility - in registers? In patient records?
Probe: What motivators are there for this documentation to be done?

4 Where does the initiative come from to do this documentation?
Probes: the people who do the documentation themselves? Their supervisors (ward-in-charge)? Their superiors (hospital managers, directors)? Outside demand (other NGO/government/UN agency eg WHO as appropriate)? Patients and their families?

5 Can you describe the importance of documentation is in this facility?
Probes: Do you think some information is given more importance or more attention in documentation than other information? Please give examples of documentation that seem to be more important and less important or not important. Why do you think this is the case? Is the information used in different ways? Is the documentation and information important to the health workers? Important to Supervisors? Important to Superiors? Important to people outside the facility? Only a task that needs to be done?

6 What is your perspective about the “culture of information and data” in this facility?
Probe: is information and data valued in this facility? How is data valued? Is an enabling environment for data recording generated or supported by unit support or hospital management / Director?

[Theme 3: INPUT - Organisational Factors]

[Training & supervision]

7 Have you ever seen any on-the-job training or supervision about documentation for the staff during the time you were working as a data collector (tracker/ clinical observer/ data verifier extractor/ supervisor)?
Probes: if yes, can you describe what did you see? How it was done? Where it was done? Who was facilitating? Who was it done for and are they the people doing the documentation? How often did you see? What was the atmosphere like – blaming? Criticising? Supportive?

[Quality]

8 Can you describe any situation you have seen health workers noticing any issues with their own documentation quality?
Probes: discrepancies between register and patient record? Missing information? Handwriting difficult to read?

9 Have you ever seen any data quality check about documentation for the staff during the time you were working as a data collector (tracker/ clinical observer/ data verifier extractor/ supervisor)?
Probes: if yes, can you describe what did you see? How it was done? Where it was done? Who did it? How often did you see? What was the atmosphere like – blaming? Criticising? Supportive?

[Availability of resources]

10 Can you describe what you have seen about the availability of the documents for mother and baby information?
Probe: Always available? Sometimes available? Can you give example of any shortage or stock-outs? If yes, how did they solve? Who solved it?

11 Can you describe other resources needed for documentation and their availability?
Probe: Examples: pens, people. Always available? Sometimes available? Can you give an example of any shortage or stock-outs? If yes, how did they solve? Who solved it?

[Theme 5: OUTPUT - Information use]

12 Can you describe any time when you saw facility staff using the data that they are collecting in these documents?
Probe: Who uses the data? What information is used? What is it used for? How often is it used? Are there some parts that are used more than others?

[Theme 6: OUTCOMES - Opportunities and recommendations]

13 In your opinion – for the recording and documentation of maternal and newborn health information how could it be improved at this facility?
Probe: training, logistics, management, supervision, monitoring, capacity development, reporting, resources etc.

14 What do you think the barriers and constraints to this improvement could be?

15 Is there anything more you would like to add about your experiences with, or views on, any of these documentation and recording processes?

Time interview end: ☐☐ : ☐☐

Thank the participant for his/her time. Remind them that the information will be kept confidential.

Interviewer comments on how the interview went:
