# Supplementary material for: Barriers and enablers to routine register data collection for newborns and mothers: EN-BIRTH multi-country validation study
Source: BMC Pregnancy Childbirth. 2021 Mar 26;21(Suppl 1):233. doi: 10.1186/s12884-020-03517-3 (PMC7995573; doi:10.1186/s12884-020-03517-3)
Supplement: Supplementary file 5 — Additional file 5. Codebook Health Workers, EN-BIRTH study. [file 12884_2020_3517_MOESM5_ESM.pdf]

SUPPLEMENT TITLE:

Every Newborn BIRTH multi-country validation study: informing measurement of coverage and quality of maternal and newborn care

PAPER TITLE:

Barriers and enablers to routine register data collection for newborns and mothers: EN-BIRTH multi-country validation study

Additional File 5: Codebook Health Workers, EN-BIRTH study

EN-BIRTH SOP – Health Worker version
Working Draft
Barriers and Enablers Qualitative – Analysis (including NVIVO)

Methodology ... 3
Qualitative data management – File Labelling ... 3
Coders ... 4
Getting started ... 4
Creating new folders and sub-nodes ... 4
Coding ... 4
Memos and annotations ... 5
Backups and data sharing ... 5
Printing ... 5
Checking coding and minimising errors ... 5
Coding frame: IDI Health Workers version ... 5
Classifications ... 6
  File Classification ... 6
  Case ... 6
General Questions ... 6
Nodes ... 7
  IMPACT - Improved health status ... 7
  INPUT - Availability of resources ... 7
  INPUT – Behavioural factors - Data Quality checking skills ... 8
  INPUT – Behavioural factors - Demand ... 9
  INPUT – Behavioural factors - Level of Knowledge of content of HIS forms ... 11
  INPUT – Behavioural factors - Motivation ... 12
  INPUT – Behavioural factors - Problem solving for HIS tasks ... 13

INPUT – Organisational factors – Finance 13
INPUT – Organisational factors – Governance 13
INPUT – Organisational factors – Planning 14
INPUT – Organisational factors - Quality 15
INPUT – Organisational factors - Supervision 15
INPUT– Organisational factors – Training 15
INPUT- Promotion of Culture of information 16
INPUT – Technical Factors - Complexity of reporting forms, procedures 16
INPUT- Technical Factors – Computer software 16
INPUT – Technical Factors – HIS Design 17
INPUT- Technical Factors – Information Technology complexity 17
OUTCOMES– Improved Health System Performance 18
OUTPUT – Improved RHIS Performance – Data quality related to information use 19
PROCESSES–RHIS Process - Data Analysis 19
PROCESSES–RHIS Process - Data Collection - When records - order 20
PROCESSES–RHIS Process - Data Collection - When records - time 21
PROCESSES–RHIS Process - Data Collection - Who records 22
PROCESSES–RHIS Process - Data Processing 22
PROCESSES–RHIS Process - Data Quality Check 22
PROCESSES–RHIS Process - Data Transmission 23
PROCESSES–RHIS Process - Feedback 23
Recommendation for improvement 23
OTHER INFORMATION 23

Methodology

Qualitative data from FGDs and in-depth and key interviews will be analysed using deductive method. Analysis will be undertaken using a framework adapted from PRISM [REF] and considering other tools [REF].

QSR International's NVivo 12 qualitative software will be used for organisation. Predetermined codes will be applied by two independent researchers in each country, data managed into units of information covering broad categories with grouping of relevant emerging themes of importance.

Qualitative data management – File Labelling

Use consistent file labelling for audio files, transcripts and translations.

Preferred format is for Tool 1 = Data Collectors

- EN-B_B&E_QualTool1_DC_XX##_site_Audio_yyyymmdd.mp4
- EN-B_B&E_QualTool1_DC_XX##_site_Transcipt_yyyymmdd.docx
- EN-B_B&E_QualTool1_DC_XX##_site_Translate_yyyymmdd.docx
- EN-B_B&E_QualTool2_DC_site _yyyymmdd.xlsx

Tool 2 identifier use: XX##_site

For Tool 3 = Health Workers

- EN-B_B&E_QualTool3_HW_ZZ##_PL_site_TTT_Audio_yyyymmdd.mp4
- EN-B_B&E_QualTool3_HW_ZZ##_PL_site_TTT_Transcipt_yyyymmdd.docx
- EN-B_B&E_QualTool3_HW_ZZ##_PL_site_TTT_Translate_yyyymmdd.docx
- EN-B_B&E_QualTool2_HW_ZZ##_PL_site_TTT_yyyymmdd.xlsx

Key

Person:	DC-XX	Data Collector	CO = Clinical Observer,<br>VE = Verifier & Extractor,<br>TR = Tracker,<br>SU = Supervisor
	HW-ZZ	Health worker	DR = Doctor, NU = Nurse
	##	use 2 digit unique identifier code number	
Place:	PL:	LD = Labour & Delivery,<br>OT = Operation Theatre,<br>NW = Neonatal Ward,<br>KM = KMC	
	Site	use site code (3-4 letters)	
Type	TTT =	FGD = Focus Group Discussion<br>IDI – In-Depth Interview	
Date:	yyyymmdd	Date of the interview	

Coders

1. In each country, the two independent coders will code separately and then need to reconcile to reach agreement.
2. Method of reconciliation – under discussion.

Getting started

3. Read through the transcript to become familiar with it and write any comments and reflections before you start coding. As you read, think about areas that were mentioned spontaneously and those that needed a lot of probing. Make comments as memos in NVIVO and include these in the report e.g. if respondents were note sure or reluctant to talk about any barriers and enablers to register documentation.
4. Only information related to the study questions will be coded.
5. The same coding frame will be used across all 5 sites to ensure consistency, but new site specific sub-nodes will be created as appropriate.
6. A separate NVIVO project template will be created for each respondent group

   Data-Collectors (Tool 1)

   Health Workers (Tool 3)

   Managers/Policy makers (Tool XX)
7. Some features of NVIVO (e.g. auto-coding) are not rigorous and will not be used for this study.

Creating new folders and sub-nodes

1. Create new sub-nodes as needed. Notify Donat and Louise Tina when there are problems/errors with the coding frame or sub-nodes that are missing and should be included for other sites. Site specific additions can be made but should be discussed with Donat and Louise Tina and will be discussed during TWG calls.
2. Interesting information that cannot be easily coded, should be coded under "OTHER – INFORMATION"
3. When creating new sub-nodes check the level of sub-node and that it is created within the appropriate node.
4. If coding into sub-nodes, check that the quote it correctly coded into the sub-node rather than the parent node. It is not necessary to code into both sub-node and parent node.

Coding

1. Code every time the respondent gives responses as this gives us an idea of how often a respondent talk about a particular theme.
2. Code longer rather than shorter sections, especially when prior sentences gives you understanding, e.g. 'she recorded it' where the previous sentence explains who 'she' is.
3. The folders are organized to limit multiple coding, although there are some places where multiple coding will occur and this is because the topic is particularly important.

Memos and annotations

1. Create memos to note your thoughts about key themes or patterns and differences in findings, for example concepts may vary by respondent characteristics (e.g. experience of healthworkers etc).
2. Use annotations to give more information that may be obtained from other parts of the interview but is not obvious from the coded quote, e.g. "that health worker" refers to "the midwife".

Backups and data sharing

1 Backup your NVIVO projects at the end of each day.

2 If working on laptop battery, save and shut down NVIVO when battery level is low as NVIVO files can become corrupt when power supply is disconnected abruptly.

Send your NVIVO files to LSHTM as agreed in the data sharing agreement. Always follow data protection SOP.

Printing

It is not usually necessary to print from NVIVO. If you need toprint the content of nodes, put your cursor in the area you want to print. Click on "File_ print", then the print options window will appear. Click "Name" and change from 'name only' to 'folder and name'. Also click "Annotations". This means that when you print, the quotes will be printed under the title for the node and any annotations will also be printed.

Checking coding and minimising errors

1. After coding each quote, check that the coding toolbar shows that the quote has been coded. Use "visualise coding bar" or "detail view" to see the coding toolbar. If a quote has been successfully coded the text in the coding toolbar will be black rather than grey and indicate which node the text has been coded at.
2. After coding each transcript, check the contents on the node to check that quotes are put in the correct folders/nodes.
3. Common errors to be aware of when coding with NVIVO include:
    - Dragging and dropping into the wrong node
    - Dragging into a folder rather than a node
    - Incorrect coding – for example coding *'who records'* as *'when records'*
    - Coding partial sentences or blank spaces

Coding frame: IDI Health Workers version

This document describes each of the nodes in the research health workers coding frame in NVIVO, they are listed in the order that they appear in the coding frame. These nodes focus on the PRISM framework and have been matrixed (see excel file "B&E DC PRISM framework_modified")

Code responses to the questions into sub-nodes if they have information relating to the red text below. A few questions relate to more than one sub-node. Code responses into *every* sub-node that is relevant – we expect some responses to be coded into 2 or more sub-nodes.

Contact Donat or Louise Tina at any time if you have a query about coding and remember to discuss as a team how you name any new sub-nodeto ensure consistency within the sites.

Classifications

File Classification

For each transcript record type of respondent here (Data Collector – Tracker, Clinical observer, Data Verifier Extractor, Supervisor, Health worker)

Case

The registers in each location are different so ensure that you classify case by “type of register”

Each site will have different names of the registers but examples may include:

- Admission register
- L&D register
- Operating Theatre register
- KMC register
- Neonatal register
- Discharge register etc.

General Questions

Question Data collector 13, 14 & 15; Health worker 15, 16, 17, 18 & 19 can be coded in any node and sub-node that is relevant.

Nodes

IMPACT - Improved health status

This does not specifically relate to any questions on Tool 1, but use this code if the respondent mentions it.

INPUT - Availability of resources

Code responses to the questions:

- *Why do you think they do it this way? What is your opinion about current documentation process and flow? Do you have what you need for documentation?*

  Probes: what are their other roles / responsibilities? Any relation to resources/logistics e.g. registers/pen/papers/copies of partograph? Where are the documents or registers situated – in one place? On different tables? What is the physical distance between the documents and the patient? How do you feel about the design of the registers/patient records/ files? Is it helpful for you? Is it clear to see when information is incomplete? Is the documentation process and flow good or not? Do the documents change? How often? Have the documents (registers, patient case records) or the documentation process changed in the last year? What are you struggling with and what helps you?
  (e.g. Data Collector Qu 1.1.3/ 1.2.3/ 1.3.3/ 1.4.3/ 1.5.3 or other place)
  (e.g. Health Worker Qu 1.1.5/1.2.5/1.3.5/1.4.5 or other place

- *Can you describe any challenges or difficulties or barriers you have observed in documentation?*

  Probes: Are there many registers or papers to fill? Do you think health workers find the documentation complicated to complete or not? Does it take a long time? Are the things they need all in one place? Do you think it is well organized?
  (e.g. Data Collector Qu 1.1.5/ 1.2.5/ 1.3.5/ 1.4.5/ 1.5.5 or other place)
  (e.g. Health Worker Qu 1.1.14/1.2.14/1.3.14/1.4.14)

- *How long does documenting usually take you? How much of your time does it take? If you divide up how much of your day is spent in documenting?*
  Probes: Try to get an estimate time e.g. number of hours or proportion of duty e.g. half of your day?
  (e.g. Health Worker Qu 1.1.8/1.2.8/1.3.8/1.4.8)

- *Can you describe what you have seen about the availability of the documents for mother and baby information?*
  Probe: Always available? Sometimes available? Can you give example of any shortage or stock-outs? If yes, how did they solve? Who solved it?
  (e.g. Data Collector *Qu 10)*
  (e.g. Health Worker Qu 8)

- *Can you describe other resources needed for documentation and their availability?*
  Probe: Examples: pens, people. Always available? Sometimes available? Can you give an example of any shortage or stock-outs? If yes, how did they solve? Who solved it?
  (e.g. Data Collector Qu 11)
  (e.g. Health Worker Qu 9)

- *There are different types of documents in L&D (e.g. clinical registers, patient case notes, patient held records, monthly summary sheets, others?) Why do we have all these different types? What are they used for?*
  Probes: - Sometimes there are formal documents and some are informal (counter books/ hand written books)? Why are there both?
  (e.g. Health Worker Qu 1.1.3/1.2.3/1.3.3/1.4.3)

- *How do you feel about the relationship of caring for the patient AND documenting your care? Which is important?*
  Probes: Why? Does documentation help care or hinder (obstruct) care?
  (e.g. Health Worker Qu 1.1.6/1.2.6/1.3.3/1.4.6)

INPUT – Behavioural factors - Competence in HIS tasks

OR

INPUT – Behavioural factors - Confidence levels for HIS tasks

Code responses to the questions in either one or both

- *Can you describe any situation you have seen health workers noticing any issues with their own documentation quality?*
  Probes: discrepancies between register and patient record? Missing information? Which parts of routine documentation are completed less well or incomplete and why? Handwriting difficult to read?
  (e.g. Data Collector Qu 8)
  (e.g. Health Worker Qu7)
- *Can you describe any time when you saw facility staff using the data that they are collecting in these documents?*
  Probe: Who uses the data? What information is used? What is it used for? How often is it used? Are there some parts that are used more than others?
  (e.g. Data Collector Qu 12)
  (e.g. Health Worker Qu14)
- Are some pieces of information (data points, data elements, indicators) recorded better than others? Can you explain why this might be?
  Probes: *give examples of data according to that ward e.g., Birth Weight, feeding practice for KMC babies*
  (e.g. Health Worker Qu 1.1.9/1.2.9/1.3.9/1.4.9)
- Information (variables) record in the different documents – which are they the same or different?
  Probes: Is everything you need to document in the register also found in the patient notes? Any variables missing? Do they overlap? Do they align? What information is typically missing?
  (e.g. Health Worker Qu 1.1.4/1.2.4/1.3.4/1.4.4)

INPUT – Behavioural factors - Data Quality checking skills

Code responses to the question:

- *Can you describe any situation you have seen health workers noticing any issues with their own documentation quality?*
  Probes: discrepancies between register and patient record? Missing information? Handwriting difficult to read?
  (e.g. Data Collector Qu 8)
  (e.g. Health Worker Qu7)
- *Information (variables) record in the different documents – which are they the same or different?*
  Probes: Is everything you need to document in the register also found in the patient notes? Any variables missing? Do they overlap? Do they align? What information is typically missing?

  (e.g. Health Worker Qu 1.1.4/1.2.4/1.3.4/1.4.4)

INPUT – Behavioural factors - Demand

Code responses to the question:

- *How do you feel about the relationship of caring for the patient AND documenting your care? Which is important?*
  Probes: Why? Does documentation help care or hinder (obstruct) care?
  (e.g. Health Worker Qu 1.1.6/1.2.6/1.3.3/1.4.6)

- *Where does the initiative come from to do this documentation?*
  Probes: the people who do the documentation themselves? Their supervisors (ward-in-charge)? Their superiors (hospital managers, directors)? Outside demand (other NGO/government/UN agency eg WHO as appropriate)? Patients and their families?
  (e.g. Data Collector Qu 4)
  (e.g. Health Worker Qu 3)

- *Can you describe the importance of documentation is in this facility?*
  Probes: Do you think some information is given more importance or more attention in documentation than other information? Please give examples of documentation that seem to be more important and less important or not important. Why do you think this is the case? Is the information used in different ways? Is the documentation and information important to the health workers? Important to Supervisors? Important to Superiors? Important to people outside the facility? Only a task that needs to be done?
  (e.g. Data collector Qu 5)
  (e.g. Health Worker Qu 4)

- *Can you describe any time when you saw facility staff using the data that they are collecting in these documents? Can you describe how this documentation is used by anyone?*

  Probe: Who uses the data? What information is used? What is it used for? How often is it used? Are there some parts that are used more than others?
  (e.g. Data Collector Qu 12)
  (e.g. Health Worker Qu 14)

- Are some pieces of information (data points, data elements, indicators) recorded better than others? Can you explain why this might be?
  Probes: give examples of data according to that ward eg Birth Weight, feeding practice for KMC babies.
  (e.g. Health Worker Qu 1.1.9/1.2.9/1.3.9/1.4.9)

- *Of the different documents that are filled in – which do you think are less or more important? I will list the documents and ask you to tell me which are the most important for 4 groups: health workers, patient, hospital management and Health Management Information System (HMIS)?*

  ***0** = not important, **1** = less important, **2** = important, **3** = more/very important, **4** = don't know*

  *Instructions – number 1 through 4 in each column. Use this table to help stimulate discussion of the respondent's perception comparing the importance using documentation for quality of patient care **versus** finances **versus** reporting, etc?*

	Name of documents	For Health care workers?	For the patient?	For hospital management?	For the HMIS?
a) Registers clinical					
b) Patient case notes (stay in facility					
c) Patient held records					
d) Monthly Summary sheets					
e) Other					
f) Other					
g) Other					
h) Other					
i) Other					
j) Other					

(e.g. Health Worker Qu 11)

- For these documents we have been talking about, what was your reasoning as to why they are more or less important? Capture some of the reasons they gave to fill the table.
  (e.g. Health Worker Qu 12)

INPUT – Behavioural factors - Level of Knowledge of content of HIS forms

Code responses to the question:

- *Can you describe any situation you have seen health workers noticing any issues with their own documentation quality?*

  Probes: discrepancies between register and patient record? Missing information? Handwriting difficult to read?
  (e.g. Data Collector Qu 8)
  (e.g. Health Worker Qu 7)

- *Are some pieces of information (data points, data elements, indicators) recorded better than others? Can you explain why this might be?*
  Probes: give examples of data according to that ward e.g., Birth Weight, feeding practice for KMC babies?
  (e.g. Health Worker Qu 1.1.9/1.2.9/1.3.9/1.4.9)

- *Information (variables) record in the different documents – which are they the same or different?*

  Probes: *Is everything you need to document in the register also found in the patient notes? Any variables missing? Do they overlap? Do they align? What information is typically missing?*
  (e.g. Health Worker Qu 1.1.4/1.2.4/1.3.4/1.4.4)

INPUT – Behavioural factors - Motivation

Code responses to the questions:

- *How do you feel about the relationship of caring for the patient AND documenting your care? Which is important?*
  ***Probes**: Why? Does documentation help care or hinder (obstruct) care?*
  (e.g. Health Worker Qu 1.1.6/1.2.6/1.3.3/1.4.6)

- *Why do you think anything is documented in this facility - in registers? In patient records?*
  Probe: What motivators are there for this documentation to be done?
  (e.g. Data Collector Qu 3)
  (e.g. Health Worker Qu 2, Qu 3)

- *Where does the initiative come from to do this documentation?*
  Probes: the people who do the documentation themselves? Their supervisors (ward-in-charge)? Their superiors (hospital managers, directors)? Outside demand (other NGO/government/UN agency eg WHO as appropriate)? Patients and their families?
  (e.g. Data Collector Qu 4)
  (e.g. Health Worker Qu 3)

- *Can you describe the importance of documentation is in this facility?*
  Probes: Do you think some information is given more importance or more attention in documentation than other information? Please give examples of documentation that seem to be more important and less important or not important. Why do you think this is the case? Is the information used in different ways? Is the documentation and information important to the health workers? Important to Supervisors? Important to Superiors? Important to people outside the facility? Only a task that needs to be done?
  (e.g. Data Collector Qu 5)
  (e.g. Health Worker Qu 4)

- *What is your perspective about the "culture of information and data" in this facility?*

  Probe: is information and data valued in this facility? How is data valued? Is an enabling environment for data recording generated or supported by unit support or hospital management / Director? Any prize/award/recognition?
  (e.g. Data Collector Qu 6)
  (e.g. Health Worker Qu 5)

- *Are some pieces of information (data points, data elements, indicators) recorded better than others? Can you explain why this might be?*
  Probes: give examples of data according to that ward e.g., Birth Weight, feeding practice for KMC babies (e.g. Health Worker Qu 1.1.9/1.2.9/1.3.9/1.4.9)

INPUT – Behavioural factors - Problem solving for HIS tasks

Code responses to the question:

- *What is your perspective about the "culture of information and data" in this facility?*

  Probe: is information and data valued in this facility? How is data valued? Is an enabling environment for data recording generated or supported by unit support or hospital management / Director?
  (e.g. Data Collector Qu 6)
  (e.g. Health Worker Qu 5)

- *Can you describe any situation you have seen health workers noticing any issues with their own documentation quality?*

  Probes: discrepancies between register and patient record? Missing information? Handwriting difficult to read?
  (e.g. Data Collector Qu 8)
  (e.g. Health Worker Qu 7)

- *Can you describe what you have seen about the availability of the documents for mother and baby information?*
  Probe: Always available? Sometimes available? Can you give example of any shortage or stock-outs? If yes, how did they solve? Who solved it?
  (e.g. Data Collector Qu 10)
  (e.g. Health Worker Qu 8)

- *Can you describe other resources needed for documentation and their availability?*
  Probe: Examples: pens, people. Always available? Sometimes available? Can you give an example of any shortage or stock-outs? If yes, how did they solve? Who solved it?
  (e.g. Data Collector Qu 11)
  (e.g. Health Worker Qu 9)

INPUT – Organisational factors – Finance

- *Can you describe what you have seen about the availability of the documents for mother and baby information?*
  Probe: Always available? Sometimes available? Can you give example of any shortage or stock-outs? If yes, how did they solve? Who solved it?
  (e.g. Data Collector Qu 10)
  (e.g. Health Worker Qu 8)

- *Can you describe other resources needed for documentation and their availability?*
  Probe: Examples: pens, people. Always available? Sometimes available? Can you give an example of any shortage or stock-outs? If yes, how did they solve? Who solved it?
  (e.g. Data Collector Qu 11)
  (e.g. Health Worker Qu 9)

INPUT – Organisational factors – Governance

- *Can you describe any situation you have seen health workers noticing any issues with their own documentation quality?*

  Probes: discrepancies between register and patient record? Missing information? Handwriting difficult to read?
  (e.g. Data Collector Qu 8)
  (e.g. Health Worker Qu 7)

- *Have you ever seen any data quality check about documentation for the staff during the time you were working as a data collector (tracker/ clinical observer/ data verifier extractor/ supervisor)?*

  Probes: if yes, can you describe what did you see? How it was done? Where it was done? Who did it? How often did you see? What was the atmosphere like – blaming? Criticising? Supportive?
  (e.g. Data Collector Qu 9)
  (e.g. Health Worker Qu 10)

INPUT – Organisational factors – Planning

Code responses to the questions:

- *Can you describe any challenges or difficulties or barriers you have observed in documentation?*

  Probes: Are there many registers or papers to fill? Do you think health workers find the documentation complicated to complete or not? Does it take a long time? Are the things they need all in one place? Do you think it is well organized?
  (e.g. Data Collector Qu 1.1.5/1.2.5/1.3.5/1.4.5/1.5.5)
  (e.g. Health Worker Qu 1.1.14/1.2.14/1.3.14/1.4.14 or other place)

- *How long does documenting usually take you? How much of your time does it take? If you divide up how much of your day is spent in documenting?*
  Probes: Try to get an estimate time e.g. number of hours or proportion of duty e.g. half of your day?
  (e.g. Health Worker Qu 1.1.8/1.2.8/1.3.8/1.4.8)

- *When patients are moved between clinical areas, have you seen any effect on documentation and recording?Example: From Antenatal ward to L&D, from L&D to OT, from OT to recovery, then to postnatal ward and discharge.*
  Probes: Do health workers hand information over verbally or written? Do the documents always stay with the patients or sometimes the patient and the notes get separated? For example: mother in OT and nurse takes partograph back to L&D to use it to write in register? Or baby goes to neonatal ward but the papers stay with the mother?
  (e.g. Data Collector Qu 2)
  (e.g. Health Worker Qu 11)

- *What is your perspective about the "culture of information and data" in this facility?*
  Probe: is information and data valued in this facility? How is data valued? Is an enabling environment for data recording generated or supported by unit support or hospital management / Director?
  (e.g. Data Collector Qu 6)
  (e.g. Health Worker Qu 5)

- *What is your opinion about their current documentation process and flow? Do you have what you need for documentation?*
  Probe: is it a good flow or not? Why do you think so? Any relation to resources/logistics e.g. registers/pen/papers/copies of partogragh? How do you feel about the design of the registers/patient records/ files? Is it helpful for you? Is it clear to see when information is incomplete? Is the documentation process and flow good or not? Do the documents change? How often? Have the documents (registers, patient case records) or the documentation process changed in the last year? What are you struggling with and what helps you?
  (e.g. *Data Collector* Qu 1.1.4/1.2.4/1.3.4/1.4.4/1.5.4)
  (e.g. Health Worker Qu 1.1.5/1.2.5/1.3.5/1.4.5)

- *There are different types of documents in L&D (e.g. clinical registers, patient case notes, patient held records, monthly summary sheets, others?) Why do we have all these different types? What are they used for?*
  Probes: - Sometimes there are formal documents and some are informal (counter books/ hand written books)? Why are there both?
  (e.g. Health Worker Qu 1.1.3/1.2.3/1.3.3/1.4.3)

- *Where does the initiative come from to do this documentation?*
  Probes: the people who do the documentation themselves? Their supervisors (ward-in-charge)? Their superiors (hospital managers, directors)? Outside demand (other NGO/government/UN agency eg WHO as appropriate)? Patients and their families?
  (e.g. Health Worker Qu 3)

INPUT – Organisational factors - Quality

- *Can you describe any situation you have seen health workers noticing any issues with their own documentation quality?*
  Probes: discrepancies between register and patient record? Missing information? Which parts of routine documentation are completed less well or incomplete and why? Handwriting difficult to read?
  (e.g. Data Collector Qu 8)
  (e.g. Health Worker Qu 7)
- Information (variables) record in the different documents – which are they the same or different?
  Probes: Is everything you need to document in the register also found in the patient notes? Any variables missing? Do they overlap? Do they align? What information is typically missing?
  (e.g. Health Worker Qu 1.1.4/1.2.4/1.3.4/1.4.4)
- Are some pieces of information (data points, data elements, indicators) recorded better than others? Can you explain why this might be?
  Probes: give examples of data according to that ward e.g., Birth Weight, feeding practice for KMC babies.
  (e.g. Health Worker Qu 1.1.9/1.2.9/1.3.9/1.4.9)

INPUT – Organisational factors - Supervision

- *Have you ever seen any on-the-job training or supervision about documentation for the staff during the time you were working as a data collector (tracker/ clinical observer/ data verifier extractor/ supervisor)?*
  Probes: if yes, can you describe what did you see? How it was done? Where it was done? Who was facilitating? Who was it done for and are they the people doing the documentation? How often did you see? What was the atmosphere like – blaming? Criticising? Supportive?
  (e.g. Data Collector Qu 7)
  (e.g. Health Worker Qu 6)

INPUT– Organisational factors – Training

- *Have you ever seen any on-the-job training or supervision about documentation for the staff during the time you were working as a data collector (tracker/ clinical observer/ data verifier extractor/ supervisor)?*
  Probes: if yes, can you describe what did you see? How it was done? Where it was done? Who was facilitating? Who was it done for and are they the people doing the documentation? How often did you see? What was the atmosphere like – blaming? Criticising? Supportive?
  (e.g. Data Collector Qu 7)
  (e.g. Health Worker Qu 6)

INPUT- Promotion of Culture of information

- *What is your perspective about the "culture of information and data" in this facility?*
  Probe: is information and data valued in this facility? How is data valued? Is an enabling environment for data recording generated or supported by unit support or hospital management / Director? Any prize/award/recognition?
  (e.g. Data Collector Qu 6)
  (e.g. Health Worker Qu 5)
- *Have you ever seen any on-the-job training or supervision about documentation for the staff during the time you were working as a data collector (tracker/ clinical observer/ data verifier extractor/ supervisor)?*
  Probes: if yes, can you describe what did you see? How it was done? Where it was done? Who was facilitating? Who was it done for and are they the people doing the documentation? How often did you see? What was the atmosphere like – blaming? Criticising? Supportive? Do you feel you have been trained appropriately, you confident? Any concerns?
  (e.g. Data Collector Qu 7)
  (e.g. Health Worker Qu 6)
- Do you ever get any feedback from the data that you send up the health system?
  Probes: What information do you receive from the HMIS? What reports? How often? Who provides the feedback? How is the feedback provided? How did you feel? What was the atmosphere like – blaming? Criticising? Supportive? Any problems? Any suggestions to improve?
  (e.g. Health Worker Qu 13)

INPUT – Technical Factors - Complexity of reporting forms, procedures

- *Can you describe any challenges or difficulties or barriers you have observed in documentation?*
  Probes: Are there many registers or papers to fill? Do you think health workers find the documentation complicated to complete or not? Does it take a long time? Are the things they need all in one place? Do you think it is well organized?
  (e.g. *Data Collector* Qu 1.1.5/1.2.5/1.3.5/1.4.5/1.5.5)
  (e.g. Health Worker Qu 1.1.14/1.2.14/1.3.14/1.4.14)
- *How long does documenting usually take you? How much of your time does it take? If you divide up how much of your day is spent in documenting?*
  Probes: Try to get an estimate time e.g. number of hours or proportion of duty e.g. half of your day?
  (e.g. Health Worker Qu 1.1.8/1.2.8/1.3.8/1.4.8)
- *What is your perspective about the "culture of information and data" in this facility?*
  Probe: is information and data valued in this facility? How is data valued? Is an enabling environment for data recording generated or supported by unit support or hospital management / Director?
  (e.g. Data Collector Qu 6)
  (e.g. Health Worker Qu 5

INPUT- Technical Factors – Computer software

- *Can you describe how routine information for mothers and babies is typically documented?*
  Probes: who does it? Who helps them? Where do they write? What documents/ registers do they fill-up? Do you see them write anywhere else (e.g. small piece of paper)? Is it all on paper or on computer or both?
  (e.g. Data Collectors Qu 1.1.1/1.2.1/1.3.1/1.4.1/1.5.1)
  (e.g. Health Worker Qu 1.1.1/1.2.1/1.3.1/1.4.1)

INPUT – Technical Factors – HIS Design

- *Can you describe how routine information for mothers and babies is typically documented?*
  Probes: who does it? Who helps them? Where do they write? What documents/ registers do they fill-up? Do you see them write anywhere else (e.g. small piece of paper)? Is it all on paper or on computer or both?
  (e.g. Data Collector Qu 1.1.1/1.2.1/1.3.1/1.4.1/1.5.1)
  (e.g. Health Worker Qu 1.1.1/1.2.1/1.3.1/1.4.1)

- *Are some pieces of information (data points, data elements, indicators) recorded better than others? Can you explain why this might be?*
  Probes: give examples of data according to that ward e.g., Birth Weight, feeding practice for KMC babies
  (e.g. Health Worker Qu 1.1.9/1.2.9/1.3.9/1.4.9)

- *Information (variables) record in the different documents – which are they the same or different?*
  Probes: Is everything you need to document in the register also found in the patient notes? Any variables missing? Do they overlap? Do they align? What information is typically missing?

- *What is your opinion about their current documentation process and flow? Do you have what you need for documentation?*
  Probe: is it a good flow or not? Why do you think so? Any relation to resources/logistics e.g. registers/pen/papers/copies of partogragh? How do you feel about the design of the registers/patient records/ files? Is it helpful for you? Is it clear to see when information is incomplete? Is the documentation process and flow good or not? Do the documents change? How often? Have the documents (registers, patient case records) or the documentation process changed in the last year? What are you struggling with and what helps you?
  (e.g. *Data Collector* Qu 1.1.4/1.2.4/1.3.4/1.4.4/1.5.4)
  (e.g. Health Worker Qu 1.1.5/1.2.5/1.3.5/1.4.5)

- *Can you describe how you feel about documentation of routine information for mothers and babies in your work place in L&D?*
  (e.g. Health Worker Qu 1.1.2/1.2.2/1.3.2/1.4.2)

INPUT- Technical Factors – Information Technology complexity

This does not specifically relate to any questions on Tool 1, but use this code if the respondent mentions it.

OUTCOMES– Improved Health System Performance

- *In your opinion – for the recording and documentation of maternal and newborn health information how could it be improved at this facility?*
  Probe: *training, logistics, management, supervision, monitoring, capacity development, reporting, resources, etc.*
  (e.g. Health Worker Qu 15)

- *What do you think the barriers and constraints to this improvement could be?*
  (e.g. Health Worker Qu 16)

- How do you feel about your role in the documentation and information about mothers and babies in your facility?
  Probe: *Is the information you collect enough?*
  (e.g. Health Worker Qu 17)

- What is your opinion to use the compiled data for mothers and babies on the wider HMIS (DHIS2) at district/national level?
  Probes: What is the role of the HMIS platform? How important is it? Does the right information go into the HMIS system from your facility? Any suggestions? Do all health workers know about the HMIS data transmission?
  (e.g. Health Worker Qu 18)

- *Is there anything else you would like to add about your experiences with, or views on, any of these documentation and recording processes?*
  (e.g. Health Worker Qu 19)
- Which is important? *Does documentation help care or hinder (obstruct) care?*
  (e.g. Health Worker Qu 1.1.6)
- *When patients are moved between clinical areas, have you seen any effect on documentation and recording?*
  Example: From Antenatal ward to L&D, from L&D to OT, from OT to recovery, then to postnatal ward and discharge.
  Probes: Do health workers hand information over verbally or written? Do the documents always stay with the patients or sometimes the patient and the notes get separated? For example: mother in OT and nurse takes partograph back to L&D to use it to write in register? Or baby goes to neonatal ward but the papers stay with the mother? How is information transported within the facility? Any problems? Any suggestions to improve?
  (e.g. Data Collector Qu 2)
  (e.g. Health Worker Qu 11)

- How is information transmitted from the facility up the health system – e.g., to the district, national level?
  Probes: Who manages/processes the information in your facility? How is information transmitted from the facility outside? How often? How do you feel about the deadlines to submit this information - is there ever any delay in transmitting this information? Any problems? Any suggestions to improve?
  (e.g. Health Worker Qu 12)

- Do you ever get any feedback from the data that you send up the health system?
  Probes: What information do you receive from the HMIS? What reports? How often? Who provides the feedback? How is the feedback provided? How did you feel? What was the atmosphere like – blaming? Criticising? Supportive? Any problems? Any suggestions to improve?
  (e.g. Health Worker Qu 13)

OUTPUT – Improved RHIS Performance – Data quality related to information use

- *Can you describe the importance of documentation is in this facility?*

  Probes: Do you think some information is given more importance or more attention in documentation than other information? Please give examples of documentation that seem to be more important and less important or not important. Why do you think this is the case? Is the information used in different ways? Is the documentation and information important to the health workers? Important to Supervisors? Important to Superiors? Important to people outside the facility? Only a task that needs to be done?
  (e.g. Data Collector Qu 5)
  (e.g. Health Worker Qu 4)

- *Can you describe any time when you saw facility staff using the data that they are collecting in these documents? Probe: Who uses the data? What information is used? What is it used for? How often is it used? Are there some parts that are used more than others?*
  (e.g. Health Worker Qu14)

PROCESSES–RHIS Process - Data Analysis

- *Can you describe any time when you saw facility staff using the data that they are collecting in these documents? Probe: Who uses the data? What information is used? What is it used for? How often is it used? Are there some parts that are used more than others?*
  (e.g. Data Collector Qu 12)
  (e.g. Health Worker Qu 14)

- Are some pieces of information (data points, data elements, indicators) recorded better than others? Can you explain why this might be?
  Probes: give examples of data according to that ward e.g., Birth Weight, feeding practice for KMC babies.
  (e.g. Health Worker Qu 1.1.9/1.2.9/1.3.9/1.4.9)

PROCESSES–RHIS Process - Data Collection - When records - order

- *Can you describe how routine information for mothers and babies is typically documented?*
  Probes: who does it? Who helps them? Where do they write? What documents/ registers do they fill-up? Do you see them write anywhere else (e.g. small piece of paper)? Is it all on paper or on computer or both?
  (e.g. Data Collector Qu 1.1.1/1.2.1/1.3.1/1.4.1/1.5.1)
  (e.g. Health Worker Qu 1.1.1 /1.2.1 /1.3.1 /1.4.1)
- *Please describe the typical documentation process (order of events) that you have seen.*
  Probes: When do they write? Relationship between care and documentation i.e. during provision of care, how does the person actually do both tasks of caring for the client AND documentation – do they do together or one before the other? If documented later, how long after the care is given?
  (e.g. Data Collector Qu 1.1.2/1.2.2/1.3.2/1.4.2/1.5.2)
  (e.g. Health Worker Qu 1.1.7/1.2.7/1.3.7/1.4.7)
- *For Health Workers, how are some specific interventions documentation - where they are documented (put a tick) and if you have any comments about the documentation of these variables?*

	a)	b)	c)	d)	e)	Comments
	Registers = L&D	Patient notes	Patient held records e.g. postnatal card	Monthly Summary s	other =	
1.Uterotonic (prophylactic)						
2.First time breast feeding						
3.Baby Resuscitation						
4.Management of neonatal infecti						
5.KMC						
6.Antenatal Corticosteroids						

- *Can you always document care immediately after it is given or sometimes does some time elapse between the care and the documentation?*
  Probe: How long for what information? What causes the delay? How do you remember the information before you write it down? What factors help or hinder the timing of documentation?
  (e.g. Health Worker Qu 1.1.10/1.2.10/1.3.10/1.4.10)

- There are different types of documents in L&D (e.g. clinical registers, patient case notes, patient held records, monthly summary sheets, others?) Why do we have all these different types? What are they used for?
  Probes: - Sometimes there are formal documents and some are informal (counter books/ hand written books)? Why are there both?
  (e.g. Health Worker Qu 1.1.3/1.2.3/1.3.3/1.4.3)

PROCESSES–RHIS Process - Data Collection - When records - time

- *Please describe the typical documentation process (order of events) that you have seen.*
  Probes: When do they write? Relationship between care and documentation i.e. during provision of care, how does the person actually do both tasks of caring for the client AND documentation – do they do together or one before the other? If documented later, how long after the care is given?
  (e.g. Data Collector Qu 1.1.2, 1.2.2, 1.3.2, 1.4.2, 1.5.2)
  (e.g. Health Worker Qu 1.1.7/1.2.7/1.3.7/1.4.7)

- Can you always document care immediately after it is given or sometimes does some time elapse between the care and the documentation?
  Probe: How long for what information? What causes the delay? How do you remember the information before you write it down? What factors help or hinder the timing of documentation
  (e.g. Health Worker Qu 1.1.10/1.2.10/1.3.10/1.4.10)

- *For Health Workers, how are some specific interventions documentation - where they are documented (put a tick) and if you have any comments about the documentation of these variables?*

	a)	b)	c)	d)	e)	Comments
	Registers = L&D	Patient notes	Patient held records e.g. postnatal card	Monthly Summary :	other =	
1.Uterotonic (prophylactic)						
2.First time breast feeding						
3.Baby Resuscitation						
4.Management of neonatal infecti						
5.KMC						
6.Antenatal Corticosteroids						

- *How long does documenting usually take you? How much of your time does it take? If you divide up how much of your day is spent in documenting?*
  Probes: Try to get an estimate time e.g. number of hours or proportion of duty e.g. half of your day?
  (e.g. Health Worker Qu 1.1.8/1.2.8/1.3.8/1.4.8)

PROCESSES–RHIS Process - Data Collection - Who records

- *Can you describe how routine information for mothers and babies is typically documented?*

  Probes: who does it? Who helps them? Where do they write? What documents/ registers do they fill-up? Do you see them write anywhere else (e.g. small piece of paper)? Is it all on paper or on computer or both?
  (e.g. Data Collector Qu 1.1.1, 1.2.1, 1.3.1, 1.4.1, 1.5.1)
  (e.g. Health Worker Qu 1.1.1/1.2.1/1.3.1/1.4.11)

PROCESSES–RHIS Process - Data Processing

- *This does not specifically relate to any questions on Tool 1, but use this code if the respondent mentions it*

PROCESSES–RHIS Process - Data Quality Check

- *Have you ever seen any data quality check about documentation for the staff during the time you were working as a data collector (tracker/ clinical observer/ data verifier extractor/ supervisor)?*
  Probes: if yes, can you describe what did you see? How it was done? Where it was done? Who did it? How often did you see? What was the atmosphere like – blaming? Criticising? Supportive?
  (e.g. Data Collector Qu 9)
  (e.g. Health Worker Qu 10)

PROCESSES–RHIS Process - Data Transmission

- *When patients are moved between clinical areas, have you seen any effect on documentation and recording? Example: From Antenatal ward to L&D, from L&D to OT, from OT to recovery, then to postnatal ward and discharge. Probes: Do health workers hand information over verbally or written? Do the documents always stay with the patients or sometimes the patient and the notes get separated? For example: mother in OT and nurse takes partograph back to L&D to use it to write in register? Or baby goes to neonatal ward but the papers stay with the mother?*
  (e.g. Data Collector Qu 2)
  (e.g. Health Worker Qu 1)
- *How is information transmitted from the facility up the health system – e.g. to the district, national level? Probes: Who manages/processes the information in your facility? How is information transmitted from the facility outside? How often? How do you feel about the deadlines to submit this information - is there ever any delay in transmitting this information? Any problems? Any suggestions to improve?*
  (e.g. Health Worker Qu 12)

PROCESSES–RHIS Process - Feedback

- *Can you describe any time when you saw facility staff using the data that they are collecting in these documents? Probe: Who uses the data? What information is used? What is it used for? How often is it used? Are there some parts that are used more than others?*
  (e.g. Data Collector Qu 12)
  (e.g. Health Worker Qu 14)
- Do you ever get any feedback from the data that you send up the health system?
  Probes: What information do you receive from the HMIS? What reports? How often? Who provides the feedback? How is the feedback provided? How did you feel? What was the atmosphere like – blaming? Criticising? Supportive? Any problems? Any suggestions to improve?
  (e.g. Health Worker Qu 13)

Recommendation for improvement

Health worker 15, 16, 17, 18 & 19 can be coded in any node and sub-node that is relevant.

OTHER INFORMATION

Interesting information that cannot be easily coded, should be coded under “OTHER – INFORMATION”
