# Supplementary material for: Barriers and enablers to routine register data collection for newborns and mothers: EN-BIRTH multi-country validation study
Source: BMC Pregnancy Childbirth. 2021 Mar 26;21(Suppl 1):233. doi: 10.1186/s12884-020-03517-3 (PMC7995573; doi:10.1186/s12884-020-03517-3)
Supplement: Supplementary file 6 — Additional file 6. Codebook Data Collectors, EN-BIRTH study. [file 12884_2020_3517_MOESM6_ESM.pdf]

SUPPLEMENT TITLE:

Every Newborn BIRTH multi-country validation study: informing measurement of coverage and quality of maternal and newborn care

PAPER TITLE:

Barriers and enablers to routine register data collection for newborns and mothers: EN-BIRTH multi-country validation study

Additional File 6: Codebook Data Collectors, EN-BIRTH study

EN-BIRTH SOP – Working Draft
Barriers and Enablers Qualitative – Analysis (including NVIVO)

EN-BIRTH SOP - DRAFT Barriers and Enablers Qualitative – Coding including NVIVO 1

Methodology 3

Qualitative data management – File Labelling 3

Coders 3

Getting started 3

Creating new folders and sub-nodes 4

Coding 5

Memos and annotations 5

Backups and data sharing 5

Printing 5

Checking coding and minimising errors 5

Coding frame: IDI Research Data Collectors 6

Classifications 6

File – Classification 6

Case – Classification 6

General Questions 6

Nodes 7

IMPACT - Improved health status 7

INPUT - Availability of resources 7

INPUT – Behavioural factors - Competence in HIS tasks 7

INPUT – Behavioural factors - Data Quality checking skills 7

INPUT – Behavioural factors - Demand 8

INPUT – Behavioural factors - Level of Knowledge of content of HIS forms 8

INPUT – Behavioural factors - Motivation 8

INPUT – Behavioural factors - Problem solving for HIS tasks 9

INPUT – Organisational factors – Finance 9
INPUT – Organisational factors – Governance 9
INPUT – Organisational factors – Planning 10
INPUT – Organisational factors - Quality 10
INPUT – Organisational factors - Supervision 10
INPUT– Organisational factors – Training 10
INPUT- Promotion of Culture of information 10
INPUT – Technical Factors - Complexity of reporting forms, procedures 11
INPUT - Technical Factors – Computer software 11
INPUT – Technical Factors – HIS Design 11
INPUT - Technical Factors – Information Technology complexity 11
OTHER INFORMATION 12
OUTCOMES – Improved Health System Performance 12
OUTPUT – Improved RHIS Performance – Data quality related to information use 12
PROCESSES – RHIS Process - Data Analysis 13
PROCESSES – RHIS Process - Data Collection - When records - order 13
PROCESSES – RHIS Process - Data Collection - When records - time 13
PROCESSES – RHIS Process - Data Collection - Who records 13
PROCESSES – RHIS Process - Data Processing 13
PROCESSES – RHIS Process - Data Quality Check 14
PROCESSES – RHIS Process - Data Transmission 14
PROCESSES – RHIS Process - Feedback 14

Methodology

Qualitative data from FGDs and in-depth and key interviews will be analysed using deductive method. Analysis will be undertaken using a framework adapted from PRISM [REF] and considering other tools [REF].

QSR International's NVivo 12 qualitative software will be used for organisation. Predetermined codes will be applied by two independent researchers in each country, data managed into units of information covering broad categories with grouping of relevant emerging themes of importance.

Qualitative data management – File Labelling

Use consistent file labelling for audio files, transcripts and translations.

Preferred format is:

- EN-B_B&E_QualTool1_DC_XX##_site_Audio_yyyymmdd.mp4
- EN-B_B&E_QualTool1_DC_XX##_site_Transcipt_yyyymmdd.docx
- EN-B_B&E_QualTool1_DC_XX##_site_Translate_yyyymmdd.docx
- EN-B_B&E_QualTool2_DC_site _yyyymmdd.xlsx

Where:
XX either: CO = Clinical Observer, VE = Verifier & Extractor, TR = Tracker, SU = Supervisor
- use 2 digit unique identifier code number
Site – use site code (3-4 letters)
yyyymmdd – date of the interview

For Tool 2 identifier use: XX##_site

Coders

1. In each country, the two independent coders will code separately and then need to reconcile to reach agreement.
2. Method of reconciliation – under discussion.

Getting started

3. Read through the transcript to become familiar with it and write any comments and reflections before you start coding. As you read, think about areas that were mentioned spontaneously and those that needed a lot of probing. Make comments as memos in NVIVO and include these in the report e.g. if respondents were note sure or reluctant to talk about any barriers and enablers to register documentation.
4. Only information related to the study questions will be coded.
5. The same coding frame will be used across all 5 sites to ensure consistency, but new site specific sub-nodes will be created as appropriate.
6. A separate NVIVO project template will be created for each respondent group

    Data-Collectors (Tool 1)

    Health Workers (Tool 3)

    Managers/Policy makers (Tool XX)

7. Some features of NVIVO (e.g. auto-coding) are not rigorous and will not be used for this study.

Creating new folders and sub-nodes

1. Create new sub-nodes as needed. Notify Donat and Louise Tina when there are problems/errors with the coding frame or sub-nodes that are missing and should be included for other sites. Site specific additions can be made but should be discussed with Donat and Louise Tina and will be discussed during TWG calls.
2. Interesting information that cannot be easily coded, should be coded under "OTHER – INFORMATION"
3. When creating new sub-nodes check the level of sub-node and that it is created within the appropriate node.
4. If coding into sub-nodes, check that the quote it correctly coded into the sub-node rather than the parent node. It is not necessary to code into both sub-node and parent node.

Coding

1. Code every time the respondent gives responses as this gives us an idea of how often a respondent talk about a particular theme.
2. Code longer rather than shorter sections, especially when prior sentences gives you understanding, e.g. 'she recorded it' where the previous sentence explains who 'she' is.
3. The folders are organized to limit multiple coding, although there are some places where multiple coding will occur and this is because the topic is particularly important.

Memos and annotations

1. Create memos to note your thoughts about key themes or patterns and differences in findings, for example concepts may vary by respondent characteristics (e.g. experience of data collectors etc).
2. Use annotations to give more information that may be obtained from other parts of the interview but is not obvious from the coded quote, e.g. "that health worker" refers to "the midwife".

Backups and data sharing

1 Backup your NVIVO projects at the end of each day.

2 If working on laptop battery, save and shut down NVIVO when battery level is low as NVIVO files can become corrupt when power supply is disconnected abruptly.

Send your NVIVO files to LSHTM as agreed in the data sharing agreement. Always follow data protection SOP.

Printing

It is not usually necessary to print from NVIVO. If you need toprint the content of nodes, put your cursor in the area you want to print. Click on "File_ print", then the print options window will appear. Click "Name" and change from 'name only' to 'folder and name'. Also click "Annotations". This means that when you print, the quotes will be printed under the title for the node and any annotations will also be printed.

Checking coding and minimising errors

1. After coding each quote, check that the coding toolbar shows that the quote has been coded. Use "visualise coding bar" or "detail view" to see the coding toolbar. If a quote has been successfully coded the text in the coding toolbar will be black rather than grey and indicate which node the text has been coded at.
2. After coding each transcript, check the contents on the node to check that quotes are put in the correct folders/nodes.
3. Common errors to be aware of when coding with NVIVO include:
    - Dragging and dropping into the wrong node
    - Dragging into a folder rather than a node
    - Incorrect coding – for example coding *'who records'* as *'when records'*
    - Coding partial sentences or blank spaces

Coding frame: IDI Research Data Collectors

This document describes each of the nodes in the IDI research data collectors coding frame in NVIVO, they are listed in the order that they appear in the coding frame. These nodes focus on the PRISM framework and have been matrixed (see excel file "B&E DC PRISM framework_modified")

Code responses to the questions into sub-nodes if they have information relating to the red text below. A few questions relate to more than one sub-node. Code responses into *every* sub-node that is relevant – we expect some responses to be coded into 2 or more sub-nodes.

Contact Donat or Louise Tina at any time if you have a query about coding and remember to discuss as a team how you name any new sub-node to ensure consistency within the sites.

Classifications

File Classification

For each transcript record type of respondent here (Data Collector – Tracker, Clinical observer, Data Verifier Extractor, Supervisor)

Case

The registers in each location are different so ensure that you classify case by "type of register"

Each site will have different names of the registers but examples may include:

- Admission register
- L&D register
- Operating Theatre register
- KMC register
- Neonatal register
- Discharge register etc.

General Questions

Question 13, 14 and 15 can be coded in any node and sub-node that is relevant

Nodes

IMPACT - Improved health status

This does not specifically relate to any questions on Tool 1, but use this code if the respondent mentions it.

INPUT - Availability of resources

Code responses to the questions:

- *Why do you think they do it this way? Probes: what are their other roles / responsibilities? Any relation to resources/logistics e.g. registers/pen/papers/copies of partograph? Where are the documents or registers situated – in one place? On different tables? What is the physical distance between the documents and the patient?*
  (*e.g.* Qu 1.1.3/ 1.2.3/ 1.3.3/ 1.4.3/ 1.5.3 or other place)
- *Can you describe any challenges or difficulties or barriers you have observed in documentation? Probes: Are there many registers or papers to fill? Do you think health workers find the documentation complicated to complete or not? Does it take a long time? Are the things they need all in one place? Do you think it is well organized?*
  (e.g. Q 1.1.5/ 1.2.5/ 1.3.5/ 1.4.5/ 1.5.5 or other place)
- *Can you describe what you have seen about the availability of the documents for mother and baby information? Probe: Always available? Sometimes available? Can you give example of any shortage or stock-outs? If yes, how did they solve? Who solved it?*
  (e.g. Q 10)
- *Can you describe other resources needed for documentation and their availability?*
  Probe: Examples: pens, people. Always available? Sometimes available? Can you give an example of any shortage or stock-outs? If yes, how did they solve? Who solved it?
  (e.g. Q11)

INPUT – Behavioural factors - Competence in HIS tasks

OR

INPUT – Behavioural factors - Confidence levels for HIS tasks

Code responses to the questions in either one or both

- *Can you describe any situation you have seen health workers noticing any issues with their own documentation quality? Probes: discrepancies between register and patient record? Missing information? Handwriting difficult to read?*
  (e.g. Qu 8)
- *Can you describe any time when you saw facility staff using the data that they are collecting in these documents? Probe: Who uses the data? What information is used? What is it used for? How often is it used? Are there some parts that are used more than others?*
  (e.g. Qu 12)

INPUT – Behavioural factors - Data Quality checking skills

Code responses to the question:

- *Can you describe any situation you have seen health workers noticing any issues with their own documentation quality? Probes: discrepancies between register and patient record? Missing information? Handwriting difficult to read?*
  (e.g. Qu 8)

INPUT – Behavioural factors - Demand

Code responses to the question:

- *Where does the initiative come from to do this documentation? Probes: the people who do the documentation themselves? Their supervisors (ward-in-charge)? Their superiors (hospital managers, directors)? Outside demand (other NGO/government/UN agency eg WHO as appropriate)? Patients and their families?*
  (e.g. Qu 4)
- *Can you describe the importance of documentation is in this facility? Probes: Do you think some information is given more importance or more attention in documentation than other information? Please give examples of documentation that seem to be more important and less important or not important. Why do you think this is the case? Is the information used in different ways? Is the documentation and information important to the health workers? Important to Supervisors? Important to Superiors? Important to people outside the facility? Only a task that needs to be done?*
  (Qu 5)
- *Can you describe any time when you saw facility staff using the data that they are collecting in these documents? Probe: Who uses the data? What information is used? What is it used for? How often is it used? Are there some parts that are used more than others?*
  (e.g. Qu 12)

INPUT – Behavioural factors - Level of Knowledge of content of HIS forms

Code responses to the question:

- *Can you describe any situation you have seen health workers noticing any issues with their own documentation quality? Probes: discrepancies between register and patient record? Missing information? Handwriting difficult to read?*
  (e.g. Qu 8)

INPUT – Behavioural factors - Motivation

Code responses to the questions:

- *Why do you think anything is documented in this facility - in registers? In patient records?*
  Probe: What motivators are there for this documentation to be done?
  (e.g. Qu 3)
- *Where does the initiative come from to do this documentation? Probes: the people who do the documentation themselves? Their supervisors (ward-in-charge)? Their superiors (hospital managers, directors)? Outside demand (other NGO/government/UN agency eg WHO as appropriate)? Patients and their families?*
  (e.g. Qu 4)
- *Can you describe the importance of documentation is in this facility? Probes: Do you think some information is given more importance or more attention in documentation than other information? Please give examples of documentation that seem to be more important and less important or not important. Why do you think this is the case? Is the information used in different ways? Is the documentation and information important to the health workers? Important to Supervisors? Important to Superiors? Important to people outside the facility? Only a task that needs to be done?*
  (Qu 5)
- *What is your perspective about the "culture of information and data" in this facility? Probe: is information and data valued in this facility? How is data valued? Is an enabling environment for data recording generated or supported by unit support or hospital management / Director?*
  (Qu 6)

INPUT – Behavioural factors - Problem solving for HIS tasks

Code responses to the question:

- *What is your perspective about the "culture of information and data" in this facility? Probe: is information and data valued in this facility? How is data valued? Is an enabling environment for data recording generated or supported by unit support or hospital management / Director?*
  (Qu 6)
- *Can you describe any situation you have seen health workers noticing any issues with their own documentation quality? Probes: discrepancies between register and patient record? Missing information? Handwriting difficult to read?*
  (e.g. Qu 8)
- *Can you describe what you have seen about the availability of the documents for mother and baby information? Probe: Always available? Sometimes available? Can you give example of any shortage or stock-outs? If yes, how did they solve? Who solved it?*
  (e.g. Q 10)
- *Can you describe other resources needed for documentation and their availability?*
  Probe: Examples: pens, people. Always available? Sometimes available? Can you give an example of any shortage or stock-outs? If yes, how did they solve? Who solved it?
  (e.g. Q11)

INPUT – Organisational factors – Finance

- *Can you describe what you have seen about the availability of the documents for mother and baby information?*
  Probe: Always available? Sometimes available? Can you give example of any shortage or stock-outs? If yes, how did they solve? Who solved it?
  (e.g. Q 10)
- *Can you describe other resources needed for documentation and their availability?*
  Probe: Examples: pens, people. Always available? Sometimes available? Can you give an example of any shortage or stock-outs? If yes, how did they solve? Who solved it?
  (e.g. Q11)

INPUT – Organisational factors – Governance

- *Can you describe any situation you have seen health workers noticing any issues with their own documentation quality? Probes: discrepancies between register and patient record? Missing information? Handwriting difficult to read?*
  (e.g. Qu 8)
- *Have you ever seen any data quality check about documentation for the staff during the time you were working as a data collector (tracker/ clinical observer/ data verifier extractor/ supervisor)? Probes: if yes, can you describe what did you see? How it was done? Where it was done? Who did it? How often did you see? What was the atmosphere like – blaming? Criticising? Supportive?*
  (e.g. Qu 9)

INPUT – Organisational factors – Planning

Code responses to the questions:

- *Why do you think they do it this way? Probes: what are their other roles / responsibilities? Any relation to resources/logistics e.g. registers/pen/papers/copies of partograph? Where are the documents or registers situated – in one place? On different tables? What is the physical distance between the documents and the patient?*
  (*e.g.* Qu 1.1.3/ 1.2.3/ 1.3.3/ 1.4.3/ 1.5.3 or other place)
- *Can you describe any challenges or difficulties or barriers you have observed in documentation? Probes: Are there many registers or papers to fill? Do you think health workers find the documentation complicated to complete or not? Does it take a long time? Are the things they need all in one place? Do you think it is well organized?*
  (e.g. Q 1.1.5/ 1.2.5/ 1.3.5/ 1.4.5/ 1.5.5)
- *When patients are moved between clinical areas, have you seen any effect on documentation and recording? Example: From Antenatal ward to L&D, from L&D to OT, from OT to recovery, then to postnatal ward and discharge. Probes: Do health workers hand information over verbally or written? Do the documents always stay with the patients or sometimes the patient and the notes get separated? For example: mother in OT and nurse takes partograph back to L&D to use it to write in register? Or baby goes to neonatal ward but the papers stay with the mother?*
  (e.g. Q 2)
- *What is your perspective about the "culture of information and data" in this facility? Probe: is information and data valued in this facility? How is data valued? Is an enabling environment for data recording generated or supported* by *unit support or hospital management / Director?*
  (Q6)

INPUT – Organisational factors - Quality

- *Can you describe any situation you have seen health workers noticing any issues with their own documentation quality? Probes: discrepancies between register and patient record? Missing information? Handwriting difficult to read?*
  (Q8)

INPUT – Organisational factors - Supervision

- *Have you ever seen any on-the-job* training *or supervision about documentation for the staff during the time you were working as a data collector (tracker/ clinical observer/ data verifier extractor/ supervisor)?*
  Probes: if yes, can you describe what did you see? How it was done? Where it was done? Who was facilitating? Who was it done for and are they the people doing the documentation? How often did you see? What was the atmosphere like – blaming? Criticising? Supportive?
  (Q 7)

INPUT– Organisational factors – Training

- *Have you ever seen any on-the-job training or supervision about documentation for the staff during the time you were working as a data collector (tracker/ clinical observer/ data verifier extractor/ supervisor)?*
  Probes: if yes, can you describe what did you see? How it was done? Where it was done? Who was facilitating? Who was it done for and are they the people doing the documentation? How often did you see? What was the atmosphere like – blaming? Criticising? Supportive?
  (Q 7)

INPUT- Promotion of Culture of information

- *What is your perspective about the "culture of information and data" in this facility? Probe: is information and data valued in this facility? How is data valued? Is an enabling environment for data recording generated or supported by unit support or hospital management / Director?*
  (Q6)

INPUT – Technical Factors - Complexity of reporting forms, procedures

- *Can you describe any challenges or difficulties or barriers you have observed in documentation? Probes: Are there many registers or papers to fill? Do you think health workers find the documentation complicated to complete or not? Does it take a long time? Are the things they need all in one place? Do you think it is well organized?*
  (e.g. Q 1.1.5/ 1.2.5/ 1.3.5/ 1.4.5/ 1.5.5)
- *What is your perspective about the "culture of information and data" in this facility? Probe: is information and data valued in this facility? How is data valued? Is an enabling environment for data recording generated or supported by unit support or hospital management / Director?*
  (Q6)

INPUT - Technical Factors – Computer software

- *Can you describe how routine information for mothers and babies is typically documented? Probes: who does it? Who helps them? Where do they write? What documents/ registers do they fill-up? Do you see them write anywhere else (e.g. small piece of paper)? Is it all on paper or on computer or both?*
  (e.g. Qu 1.1.1, 1.2.1, 1.3.1, 1.4.1, 1.5.1)

INPUT – Technical Factors – HIS Design

- *Can you describe how routine information for mothers and babies is typically documented? Probes: who does it? Who helps them? Where do they write? What documents/ registers do they fill-up? Do you see them write anywhere else (e.g. small piece of paper)? Is it all on paper or on computer or both?*
  (e.g. Qu 1.1.1, 1.2.1, 1.3.1, 1.4.1, 1.5.1)
- *What is your opinion about their current documentation process and flow? Probe: is it a good flow or not? Why do you think so?*
  (e.g. Q 1.1.4/ 1.2.4/ 1.3.4/ 1.4.4/ 1.5.4)

INPUT - Technical Factors – Information Technology complexity

This does not specifically relate to any questions on Tool 1, but use this code if the respondent mentions it.

OTHER INFORMATION

Interesting information that cannot be easily coded, should be coded under "OTHER – INFORMATION"

OUTCOMES – Improved Health System Performance

This does not specifically relate to any questions on Tool 1, but use this code if the respondent mentions it.

OUTPUT – Improved RHIS Performance – Data quality related to information use

- *Can you describe the importance of documentation is in this facility? Probes: Do you think some information is given more importance or more attention in documentation than other information? Please give examples of documentation that seem to be more important and less important or not important. Why do you think this is the case? Is the information used in different ways? Is the documentation and information important to the health workers? Important to Supervisors? Important to Superiors? Important to people outside the facility? Only a task that needs to be done?*
  (Qu 5)

PROCESSES – RHIS Process - Data Analysis

- *Can you describe any time when you saw facility staff using the data that they are collecting in these documents? Probe: Who uses the data? What information is used? What is it used for? How often is it used? Are there some parts that are used more than others?*
  (e.g. Qu 12)

PROCESSES – RHIS Process - Data Collection - When records - order

- *Can you describe how routine information for mothers and babies is typically documented? Probes: who does it? Who helps them? Where do they write? What documents/ registers do they fill-up? Do you see them write anywhere else (e.g. small piece of paper)? Is it all on paper or on computer or both?*
  (e.g. Qu 1.1.1, 1.2.1, 1.3.1, 1.4.1, 1.5.1)
- *Please describe the typical documentation process (order of events) that you have seen. Probes: When do they write? Relationship between care and documentation i.e. during provision of care, how does the person actually do both tasks of caring for the client AND documentation – do they do together or one before the other? If documented later, how long after the care is given?*
  (e.g. Qu 1.1.2, 1.2.2, 1.3.2, 1.4.2, 1.5.2)
- *We are not asking you specifically about what is written in the routine facility registers /documents, but we are interested if you observed anything about some specific intervention documentation - Who documented? How did they document? When did they document?*
  - *The interventions of interest are:*
    Antenatal Corticosteroids? (e.g. Q1.1.6.1)
    Uterotonic (prophylactic)? (e.g. Q1.2.6.1 and 1.3.6)
    Essential Newborn Care (first time breast feeding)? (e.g. Q1.2.6.2 and 1.3.6.2)
    Resuscitation of the baby? (e.g. Q1.2.6.3 and 1.3.6.3)
    Antenatal Corticosteroids? (e.g. Q1.2.6.4 and 1.3.6.4
    Management of neonatal infection? (e.g. Q1.4.6.1 and 1.5.6.1)

PROCESSES – RHIS Process - Data Collection - When records - time

- *Please describe the typical documentation process (order of events) that you have seen. Probes: When do they write? Relationship between care and documentation i.e. during provision of care, how does the person actually do both tasks of caring for the client AND documentation – do they do together or one before the other? If documented later, how long after the care is given?*
  (e.g. Qu 1.1.2, 1.2.2, 1.3.2, 1.4.2, 1.5.2)
- *We are not asking you specifically about what is written in the routine facility registers /documents, but we are interested if you observed anything about some specific intervention documentation - Who documented? How did they document? When did they document?*
  - *The interventions of interest are:*
    Antenatal Corticosteroids? (e.g. Q1.1.6.1)
    Uterotonic (prophylactic)? (e.g. Q1.2.6.1 and 1.3.6)
    Essential Newborn Care (first time breast feeding)? (e.g. Q1.2.6.2 and 1.3.6.2)
    Resuscitation of the baby? (e.g. Q1.2.6.3 and 1.3.6.3)
    Antenatal Corticosteroids? (e.g. Q1.2.6.4 and 1.3.6.4
    Management of neonatal infection? (e.g. Q1.4.6.1 and 1.5.6.1)

PROCESSES – RHIS Process - Data Collection - Who records

- *Can you describe how routine information for mothers and babies is typically documented? Probes: who does it? Who helps them? Where do they write? What documents/ registers do they fill-up? Do you see them write anywhere else (e.g. small piece of paper)? Is it all on paper or on computer or both?*
  (e.g. Qu 1.1.1, 1.2.1, 1.3.1, 1.4.1, 1.5.1)

PROCESSES – RHIS Process - Data Processing

- *This does not specifically relate to any questions on Tool 1, but use this code if the respondent mentions it*

PROCESSES – RHIS Process - Data Quality Check

- *Have you ever seen any data quality check about documentation for the staff during the time you were working as a data collector (tracker/ clinical observer/ data verifier extractor/ supervisor)?*
  Probes: if yes, can you describe what did you see? How it was done? Where it was done? Who did it? How often did you see? What was the atmosphere like – blaming? Criticising? Supportive
  (e.g. Q9)

PROCESSES – RHIS Process - Data Transmission

- *When patients are moved between clinical areas, have you seen any effect on documentation and recording?*
  Example: From Antenatal ward to L&D, from L&D to OT, from OT to recovery, then to postnatal ward and discharge.
  Probes: Do health workers hand information over verbally or written? Do the documents always stay with the patients or sometimes the patient and the notes get separated? For example: mother in OT and nurse takes partograph back to L&D to use it to write in register? Or baby goes to neonatal ward but the papers stay with the mother?
  (e.g. Q 2)

PROCESSES – RHIS Process - Feedback

- *Can you describe any time when you saw facility staff using the data that they are collecting in these documents?*
  Probe: Who uses the data? What information is used? What is it used for? How often is it used? Are there some parts that are used more than others?
  (e.g. Qu 12)
