# Supplementary material for: Barriers and enablers to routine register data collection for newborns and mothers: EN-BIRTH multi-country validation study
Source: BMC Pregnancy Childbirth. 2021 Mar 26;21(Suppl 1):233. doi: 10.1186/s12884-020-03517-3 (PMC7995573; doi:10.1186/s12884-020-03517-3)
Supplement: Supplementary file 9 — Additional file 9. Labour and Delivery Registers, formal and informal, EN-BIRTH study. [file 12884_2020_3517_MOESM9_ESM.pdf]

SUPPLEMENT TITLE:

Every Newborn BIRTH multi-country validation study: informing measurement of coverage and quality of maternal and newborn care

PAPER TITLE:

Barriers and enablers to routine register data collection for newborns and mothers: EN-BIRTH multi-country validation study

Additional File 9: Labour and Delivery Registers, formal and informal, EN-BIRTH study

	**Azimpur Tertiary**	**Kushtia District**	**Pokhara Regional**	**Temeke Regional**	**Muhimbili National**
**Formal register (printed)**	Labour and Delivery<br>Conduction	Labour and Delivery<br>Conduction<br>KMC	Delivery<br>Admission<br>Discharge<br>Birth Card	Delivery	Delivery
**Informal register (hand-written)**	Admission<br>Child<br>Child<br>Neonatal	Admission<br>Child	1st stage round<br>ANC round<br>PNC round<br>Inventory record	Admission<br>Score Sheet<br>Special Book	Perinatal (midwifery)<br>Admission<br>Neonatal<br>OT<br>Referral<br>Transfer
