# Supplementary material for: Barriers and enablers to routine register data collection for newborns and mothers: EN-BIRTH multi-country validation study
Source: BMC Pregnancy Childbirth. 2021 Mar 26;21(Suppl 1):233. doi: 10.1186/s12884-020-03517-3 (PMC7995573; doi:10.1186/s12884-020-03517-3)
Supplement: Supplementary file 10 — Additional file 10. Labour ward routine register column design for maternal and newborn indicators, EN-BIRTH study. [file 12884_2020_3517_MOESM10_ESM.pdf]

SUPPLEMENT TITLE:

Every Newborn BIRTH multi-country validation study: informing measurement of coverage and quality of maternal and newborn care

PAPER TITLE:

Barriers and enablers to routine register data collection for newborns and mothers: EN-BIRTH multi-country validation study

Additional File 10: Labour ward routine register column design for maternal and newborn indicators, EN-BIRTH study

	**BD - Azimpur Tertiary**		**BD - Kushtia District**		**NP - Pokhara Regional**	**TZ - Temeke District**	**TZ - Muhimbili Regional**
	**Original register**	**Revised register**	**Original register**	**Revised register**			
**Labour and Delivery ward**							
**1. Uterotonic for 3rd Stage Labour**							
**Register design:** Column allotted data element	non-specific	specific column	non-specific	specific column	no column	specific 2 columns	specific 2 columns
Column 1 heading	drugs given	AMTSL (footnote oxytocin)	Drugs given	AMTSL (footnote oxytocin)		Mother given uterotonic	Mother given uterotonic
Column 1: data element completed if uterotonic given	"NVD drugs given"	tick	drug name	tick		yes (in Swahili)	yes (in Swahili)
Column 1: data element completed if uterotonic not given	blank	blank (clear instruction)	blank	blank (clear instruction)		no (in Swahili)	no (in Swahili)
Column 2 heading						AMSTL	AMSTL
Column 2: data element completed if uterotonic given						O (=oxytocin)<br>E (= ergometrine)<br>M (= misosprostol)	O (=oxytocin)<br>E (= ergometrine)<br>M (= misosprostol)
Column 2: data element completed if uterotonic not given						dash or No (in Swahili)	dash or No (in Swahili)
**2. Early Initiation of Breast Feeding**							
**Register design:** Column allotted data element	no column	specific column	non-specific	specific column	no column	specific 2 columns	specific 2 columns
Column 1 heading		Breast fed within 1 hour	Newborn	Breast fed within 1 hour		Breast fed within 1 hour	Breast fed within 1 hour
Column 1: data element completed if breastfed		tick	Breastfed	tick		Yes (in Swahili)	Yes (in Swahili)
Column 1: data element completed if not breastfed		blank (clear instruction)	Blank	blank (clear instruction)		No (in Swahili)	No (in Swahili)
Column 2 heading						Feeding Baby	Feeding Baby
Column 2: data element completed if breastfed						EBF (= breast milk)<br>RF (= other milk)	EBF (= breast milk)<br>RF (= other milk)
Column 2: data element completed if not breastfed						dash or No (in Swahili)	dash or No (in Swahili)
**3. Neonatal Resuscitation - Bag-mask-ventilation**							
**Register design:** Column allotted data element	no column	specific column	no column	specific column	no column	specific column	specific column
Column heading		Bag Mask Ventilated		Bag Mask Ventilated		Helping Babies Breathe	Helping Babies Breathe
Data element completed if Bag Mask Ventilated		Tick		Tick		3 (= BMV)	3 (= BMV)
Data element completed if not Bag Mask Ventilated		blank (clear instruction)		blank (clear instruction)		No (in Swahili)	No (in Swahili)
