# Supplementary material for: Barriers and enablers to routine register data collection for newborns and mothers: EN-BIRTH multi-country validation study
Source: BMC Pregnancy Childbirth. 2021 Mar 26;21(Suppl 1):233. doi: 10.1186/s12884-020-03517-3 (PMC7995573; doi:10.1186/s12884-020-03517-3)
Supplement: Supplementary file 11 — Additional file 11. Demographic characteristics of respondents for barriers and enablers objective, labour/newborn wards, EN-BIRTH study. [file 12884_2020_3517_MOESM11_ESM.pdf]

SUPPLEMENT TITLE:

Every Newborn BIRTH multi-country validation study: informing measurement of coverage and quality of maternal and newborn care

PAPER TITLE:

Barriers and enablers to routine register data collection for newborns and mothers: EN-BIRTH multi-country validation study

Additional File 11: Demographic characteristics of respondents for barriers and enablers objective, labour/ newborn wards, EN-BIRTH study

Category	Details	Azimpur				Kushtia				Pokhara				Temeke				Muhimbili			
		In-Depth interview			FGD	In-Depth interview			FGD	In-Depth interview			FGD	In-Depth interview			FGD	In-Depth interview			FGD
		Data-collector	Nurse-midwives	Doctors	Nurse-midwives	Data-collector	Nurse-midwives	Doctors	Nurse-midwives	Data-collector	Nurse-midwives	Doctors	Nurse-midwives	Data-collector	Nurse-midwives	Doctors	Nurse-midwives	Data-collector	Nurse-midwives	Doctors	Nurse-midwives
Hospital Healthworker	Nurse midwives	-	6	-	5	-	4	-	8	-	6	-	6	-	6	-	6	-	8	-	7
	Medical doctors	-	-	2	-	-	-	2	-	-	-	2	-	-	-	2	-	-	-	2	-
EN-BIRTH Researchers	Supervisors	1	-	-	-	1	-	-	-	3	-	-	-	2	-	-	-	2	-	-	-
	Clinical Observers	4	-	-	-	4	-	-	-	8	-	-	-	4	-	-	-	4	-	-	-
	Data extractors /verifiers	1	-	-	-	1	-	-	-	3	-	-	-	4	-	-	-	4	-	-	-
	Trackers	4	-	-	-	4	-	-	-	3	-	-	-	4	-	-	-	4	-	-	-
Age	19-29 years	8	0	1	0	7	0	0	0	16	3	0	0	11	3	0	1	11	2	0	3
	30-39 years	2	2	0	0	3	1	1	3	1	3	1	1	2	1	1	1	3	4	2	4
	40-49 years	0	1	0	1	0	3	0	4	0	0	1	5	1	0	1	2	0	1	0	0
	50-59 years	0	3	1	4	0	0	1	1	0	0	0	0	0	2	0	2	0	1	0	0
Sex	Female	7	6	2	5	6	4	1	8	17	6	1	6	7	5	0	5	8	7	1	5
	Male	3	0	0	0	4	0	1	0	0	0	1	0	7	1	2	1	6	1	1	2
Education	Certificate	2	1	0	5	1	0	0	1	13	1	0	2	4	4	0	4	4	0	0	0
	Diploma	3	4	0	0	5	4	0	6	0	0	0	0	10	2	0	2	9	4	0	3
	Degree	5	1	2	0	4	0	2	1	4	5	2	4	0	0	2	0	1	4	2	4
Average time worked in ward/months	Labour ward	12	15	15	-	12	14	6	-	5.7	5.5	6	-	3.5	2.3	2.2	-	2.5	2.1	6	-
	Operating theatre	12	14	0	-	12	12	0	-	3.5	1.8	6	-	2.5	2.6	2.2	-	2.1	1.6	6	-
	Neonatal ward	12	5	2	-	12	12	9	-	3.5	7.5	0	-	2.25	1.6	1.2	-	2	1.2	5	-
	KMC ward	12	5	0	-	12	4	0	-	1.5	0	0	-	3	8	1.2	-	1.9	2.5	5	-
