# Supplementary material for: Barriers and enablers to routine register data collection for newborns and mothers: EN-BIRTH multi-country validation study
Source: BMC Pregnancy Childbirth. 2021 Mar 26;21(Suppl 1):233. doi: 10.1186/s12884-020-03517-3 (PMC7995573; doi:10.1186/s12884-020-03517-3)

SUPPLEMENT TITLE:

Every Newborn BIRTH multi-country validation study: informing measurement of coverage and quality of maternal and newborn care

PAPER TITLE:

Barriers and enablers to routine register data collection for newborns and mothers: EN-BIRTH multi-country validation study

Additional File 15: Labour ward register order within all documentation, by indicator - data collector respondents, EN-BIRTH study

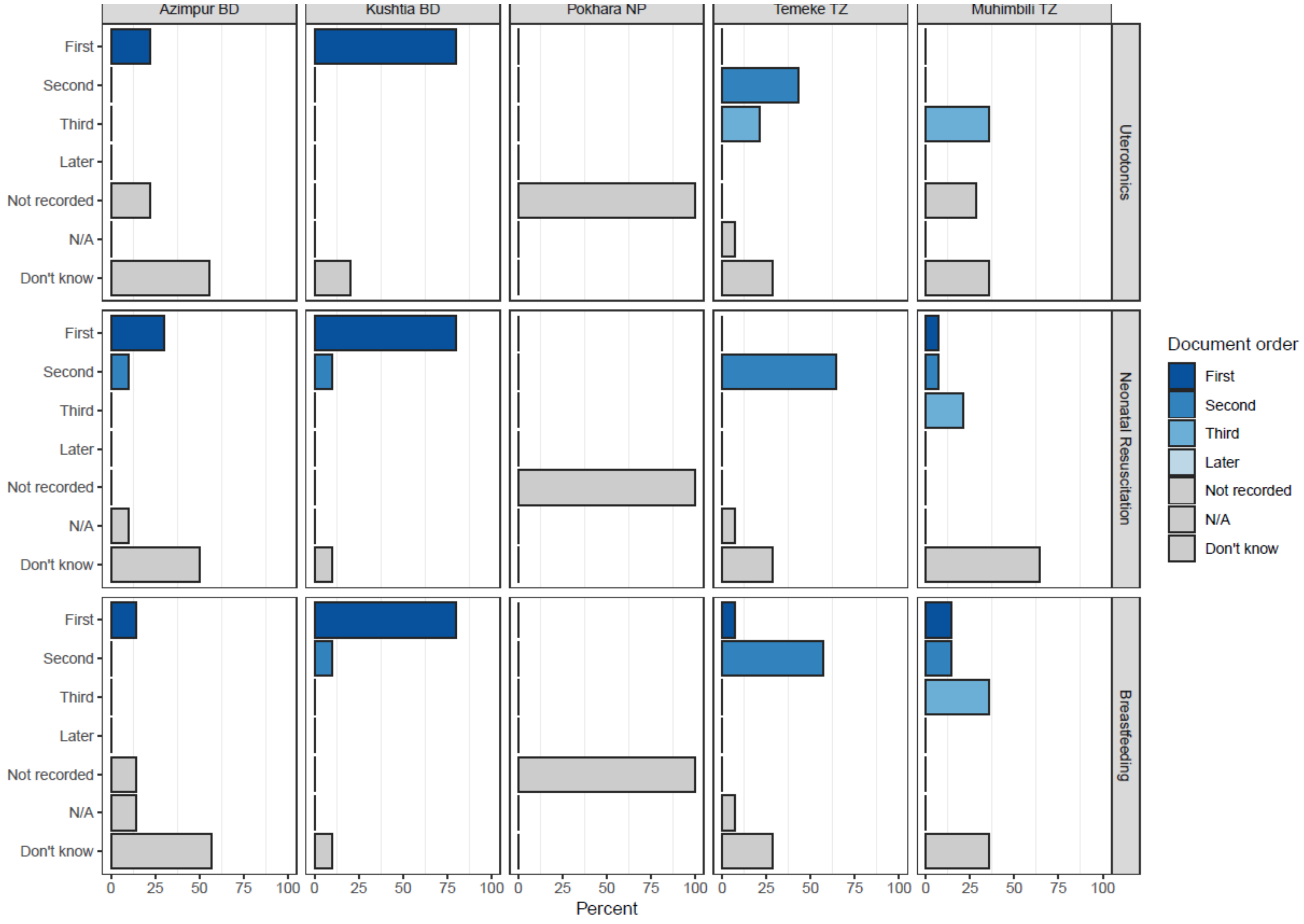

Supplement: Supplementary file 15 — Additional file 15. Labour ward register order within all documentation, by indicator - data collector respondents, EN-BIRTH study. [file 12884_2020_3517_MOESM15_ESM.pdf]
