# Supplementary material for: Barriers and enablers to routine register data collection for newborns and mothers: EN-BIRTH multi-country validation study
Source: BMC Pregnancy Childbirth. 2021 Mar 26;21(Suppl 1):233. doi: 10.1186/s12884-020-03517-3 (PMC7995573; doi:10.1186/s12884-020-03517-3)
Supplement: Supplementary file 16 — Additional file 16. Estimated minutes between care and documentation by indicator, care-documentation checklist, EN-BIRTH study. [file 12884_2020_3517_MOESM16_ESM.pdf]

SUPPLEMENT TITLE:

Every Newborn BIRTH multi-country validation study: informing measurement of coverage and quality of maternal and newborn care

PAPER TITLE:

Barriers and enablers to routine register data collection for newborns and mothers: EN-BIRTH multi-country validation study

Additional File 16: Estimated minutes between care and documentation by indicator, care-documentation checklist, EN-BIRTH study

		Estimated time in minutes between care and documentation					
	**Indicator**	Azimpur BD Tertiary	Kushtia BD District	Pokhara NP Regional	Temeke TZ Regional	Muhimbili TZ National	All hospitals
**Health workers**	Uterotonics	11	26	N/A	39	16	23
	Neonatal Resuscitation	11	16	N/A	23	18	17
	Breastfeeding	9	18	N/A	22	28	19
	All indicator hospital average	10	20	N/A	28	20	19
**Data collectors**	Uterotonics	9	27	N/A	37	31	26
	Neonatal Resuscitation	9	19	N/A	26	32	21
	Breastfeeding	8	22	N/A	26	39	24
	All indicator hospital average	9	22	N/A	30	34	24
