# Supplementary material for: Barriers and enablers to routine register data collection for newborns and mothers: EN-BIRTH multi-country validation study
Source: BMC Pregnancy Childbirth. 2021 Mar 26;21(Suppl 1):233. doi: 10.1186/s12884-020-03517-3 (PMC7995573; doi:10.1186/s12884-020-03517-3)
Supplement: Supplementary file 17 — Additional file 17. Ethical approval of local institutional review boards for EN-BIRTH study. [file 12884_2020_3517_MOESM17_ESM.pdf]

SUPPLEMENT TITLE:

Every Newborn BIRTH multi-country validation study: informing measurement of coverage and quality of maternal and newborn care

PAPER TITLE:

Barriers and enablers to routine register data collection for newborns and mothers: EN-BIRTH multi-country validation study

Additional File 17: Ethical approval of local institutional review boards, EN-BIRTH study

Country	Institutional Review Boards	Date	Number/Ref
UK	London School of Hygiene & Tropical Medicine (LSHTM) Interventions Research Ethics Committee	03/10/16	11780
Bangladesh	Icddr,b Research Review Committee<br>Icddr,b Ethical Review Committee	11/08/16<br>14/11/16	PR 16055
Nepal	Nepal Health Research Council (NHRC)	08/08/16	187/2016
Tanzania	National Institute for Medical Research (NIMRI)	20/01/17	NIMR/HQ/R.8a/Vol IX/2394
	Ifakara Health Institute	20/10/16	IHI/IRB/No: 032-2016
	Muhimbili University of Health and Allied Sciences research and Publications committee	21/10/16	2016-10-21-/AEC/Vol.XI/310
